# Notch3 Interactome Analysis Identified WWP2 as a Negative Regulator of Notch3 Signaling in Ovarian Cancer

**DOI:** 10.1371/journal.pgen.1004751

**Published:** 2014-10-30

**Authors:** Jin-Gyoung Jung, Alexander Stoeck, Bin Guan, Ren-Chin Wu, Heng Zhu, Seth Blackshaw, Ie-Ming Shih, Tian-Li Wang

**Affiliations:** 1 Departments of Pathology and Gynecology/Obstetrics, The Johns Hopkins University School of Medicine, Baltimore, Maryland, United States of America; 2 Department of Pathology, Chang Gung Memorial Hospital and Chang Gung University School of Medicine, Taoyuan, Taiwan; 3 Department of Pharmacology, The Johns Hopkins University School of Medicine, Baltimore, Maryland, United States of America; 4 Center for High-Throughput Biology, The Johns Hopkins University School of Medicine, Baltimore, Maryland, United States of America; 5 Department of Neuroscience, The Johns Hopkins University School of Medicine, Baltimore, Maryland, United States of America; 6 Institute for Cell Engineering, Johns Hopkins University School of Medicine, Baltimore, Maryland, United States of America; 7 Department of Oncology, The Johns Hopkins University School of Medicine, Baltimore, Maryland, United States of America; St Jude Children's Research Hospital, United States of America

## Abstract

The Notch3 signaling pathway is thought to play a critical role in cancer development, as evidenced by the *Notch3* amplification and rearrangement observed in human cancers. However, the molecular mechanism by which Notch3 signaling contributes to tumorigenesis is largely unknown. In an effort to identify the molecular modulators of the Notch3 signaling pathway, we screened for Notch3-intracellular domain (N3-ICD) interacting proteins using a human proteome microarray. Pathway analysis of the Notch3 interactome demonstrated that ubiquitin C was the molecular hub of the top functional network, suggesting the involvement of ubiquitination in modulating Notch3 signaling. Thereby, we focused on functional characterization of an E3 ubiquitin-protein ligase, WWP2, a top candidate in the Notch3 interactome list. Co-immunoprecipitation experiments showed that WWP2 interacted with N3-ICD but not with intracellular domains from other Notch receptors. Wild-type WWP2 but not ligase-deficient mutant WWP2 increases mono-ubiquitination of the membrane-tethered Notch3 fragment, therefore attenuating Notch3 pathway activity in cancer cells and leading to cell cycle arrest. The mono-ubiquitination by WWP2 may target an endosomal/lysosomal degradation fate for Notch3 as suggested by the fact that the process could be suppressed by the endosomal/lysosomal inhibitor. Analysis of The Cancer Genome Atlas dataset showed that the majority of ovarian carcinomas harbored homozygous or heterozygous deletions in WWP2 locus, and there was an inverse correlation in the expression levels between WWP2 and Notch3 in ovarian carcinomas. Furthermore, ectopic expression of WWP2 decreased tumor development in a mouse xenograft model and suppressed the Notch3-induced phenotypes including increase in cancer stem cell-like cell population and platinum resistance. Taken together, our results provide evidence that WWP2 serves as a tumor suppressor by negatively regulating Notch3 signaling in ovarian cancer.

## Author Summary

Notch pathway is important for many cellular activities, and its dysregulation leads to several diseases in humans, including cancer. Although Notch hyperactivity has been observed in many types of cancers, the interactome of Notch receptor remains largely unknown, especially for Notch3, which is involved in ovarian cancer pathogenesis. This article is the first study, to our knowledge, that delineates the Notch3 interacting network, and demonstrates that one of the Notch3 interacting proteins, WWP2, an E3 ubiquitin-protein ligase, plays a major role in negative regulation of Notch3 signaling in cancer cells. WWP2 locus was found to be deleted, and its mRNA down-regulated in a significant fraction of ovarian carcinomas. Ectopic expression of WWP2 reduced tumorigenicity of ovarian cancer cells, and counteracted Notch3-mediated phenotypes, including promotion of cancer stem-like cell phenotype and platinum resistance, further supporting its tumor suppressor role. The results from this study provide new insights into how Notch3 signaling contributes to cancer development, and should have implications for the design of Notch3-based cancer therapy.

## Introduction

Notch signaling is a highly conserved cell-cell communication system present in multicellular organisms, and has been shown to be involved in cell fate decision, cell lineage specification, cell proliferation, and survival. Dysregulation of Notch signaling has been known to play a significant role in many diseases including cancer. However, its role as either an oncogene or a tumor suppressor is context- or tissue-type dependent, as both activating mutations and inactivating mutations have been identified in human cancers of different tissue lineages.

In contrast to Drosophila, which has only one type of Notch receptor, mammals contain four types of Notch receptors (Notch1, Notch2, Notch3, and Notch4), perhaps having evolved to support the demands of intricate signaling networks in higher order organisms. The four Notch receptors in mammals share several conserved protein structures which include a large ligand-binding extracellular fragment containing EGF-like repeats, followed by a membrane spanning region and an intracellular fragment containing a RAM domain, tandem ankyrin repeats, and a PEST domain. Notch signaling is initiated by ligand-receptor engagement, which triggers sequential juxta-plasma membrane protein cleavages by ADAM and γ-secretase proteases, leading to the release of intracellular cytoplasmic domain (ICD) fragments. The ICD is then translocated into the nucleus where it acts as a coactivator of RBPJ (CSL)-dependent gene expression. Notch-dependent transcription can be regulated by the quantity of ligand and receptor present on the cell surface, binding affinity of the receptor for its ligand, and protein stability of the ICD.

It has been thought that different Notch receptors share canonical functions but also differentially regulate cellular signaling. Although members of Notch receptor may complement the function of another in some circumstances, each receptor exhibits unique, context-dependent functions. For example, Notch1 receptor knockout causes an embryonically lethality in mice, while Notch3 receptor knockout does not cause the same lethality although the Notch3-null mice harbor minor, yet appreciable, postnatal defects in smooth muscle structure and vascular function [1], [2]. Notch3, but not other Notch receptors, are highly expressed in arterial smooth muscle cells and may regulate differentiation of arterial smooth muscle cells and capillary growth through a paracrine interaction between smooth muscle cells and endothelial cells. Mutations of single amino acid residues at the EGF-like domain of Notch3 affect the functions of vascular smooth muscle cells, leading to apoptosis and degeneration of these cells, and resulting in brain arteriopathy, recurrent strokes, and other symptoms of cerebral autosomal dominant arteriopathy with sub-cortical infarcts and leukoencephalopathy, (CADASIL) [3]. *Notch3* gene amplification occurs in ovarian carcinoma, and rearrangement occurs in a subset of malignant glomus tumors [4], [5]. Recent large-scale genomic and expression analysis in ovarian high-grade serous carcinomas (HGSC) by the TCGA consortium has further confirmed the frequent amplification of the *Notch3* locus and other aberrations including gene amplification and transcriptional upregulation in members of Notch signaling pathways [6]. Notch3 has been shown to be essential for tumor growth and development of drug resistance [7]. Therefore, targeting the Notch3 pathway represents a promising therapeutic strategy for cancers with dysregulated Notch3 signaling.

Despite the important roles of Notch3 in human diseases, the molecules that mediate or modulate Notch3 signaling remain to be identified. To this end, we have previously performed a systems biology approach using ChIP-chip and transcriptome analyses to identify Notch3 direct target genes in cancer cells [8]. The current study was aimed at identifying Notch3-binding proteins with the presumption that those proteins may modulate Notch3 function. We used a human proteome microarray to screen for N3-ICD protein binding partners and identified an array of Notch3-interacting proteins. Network analysis of the newly identified Notch3 interactome indicated the involvement of ubiquitination in regulating Notch3 signaling; therefore we performed functional studies to characterize an E3-ubiquitin ligase, WWP2, and demonstrated that it is a negative regulator of Notch3 in ovarian cancer.

## Results

### Identification of Notch3 interactome

**Figure 1:**
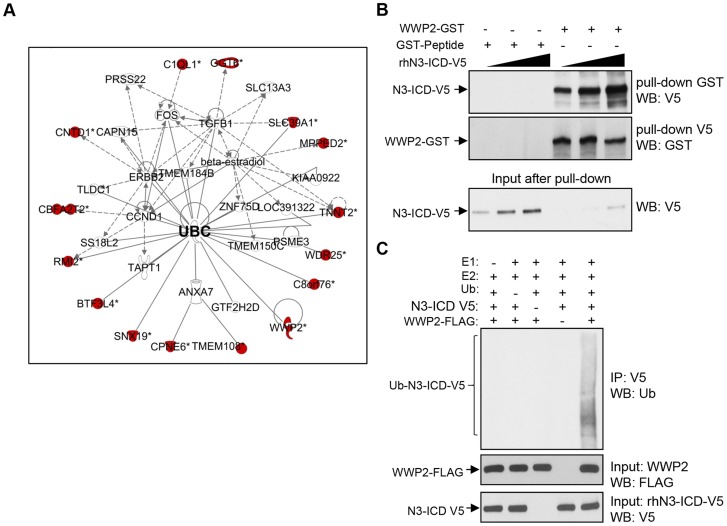
Identification of Notch3 interacting proteins in protein array. (**A**) Ingenuity pathway analysis shows that Notch3 interaction proteins are enriched in a network involved in gene expression, cell cycle, and development. Colored symbols: Notch3-interacting proteins identified in our screen. UBC: ubiquitin C. (**B**) Western blot analysis of GST pull-down was performed with GST-peptide control or recombinant WWP2-GST in the presence of increasing concentrations of rhN3-ICD protein using anti-GST antibody or anti V5 antibody. Lower panel shows western blot analysis of flow through after GST pull-down. Reduced N3-ICD is seen only after pulldown with WWP2-GST but not with control GST-peptide. (**C**) In vitro ubiquitination experiment was performed using recombinant human Notch3 as a substrate in the presence of recombinant ubiquitin, E1 (UBE1), E2 (UbcH5B), and FLAG-tagged WWP2. Ubiquitinated species of rhN3-ICD were detected by immunoprecipitation with V5 antibody followed by western blot with anti-ubiquitin antibody (upper panel). Western blot analysis with anti-FLAG antibody or anti-V5 antibody was performed to detect total input amounts of WWP2 and N3-ICD (lower panels). Ubiquitinated rhN3-ICD was only detected when WWP2 was present.

The purpose of this study was to identify and characterize proteins that interacted with the Notch3 intracellular cytoplasmic domain (N3-ICD). First, we expressed and purified human recombinant N3-ICD protein tagged with the V5 epitope (rhN3-ICD-V5). The rhN3-ICD-V5 protein was then used as a “bait” to screen for binding proteins on the human proteome microarray, comprised of 16,368 individually purified, full-length human proteins [9]. This screen identified a number of N3-ICD-interacting proteins. Among them, RBPJ (CSL), a well-known transcriptional co-factor of the Notch receptors, was at the top of the N3-ICD binding partner list and displayed a high fluorescent binding score (Table S1). This observation indicated that protein microarray is a valid approach to rapidly and reliably identify Notch3 interacting proteins. We next performed Ingenuity Pathway Analysis to search for enriched functional links among the newly identified N3-ICD interactome. The results demonstrated that the enriched functional networks include gene expression, cell cycle, cell signaling, and cellular development (Table S2). As illustrated in Fig. 1A, ubiquitin C (UBC) is a major hub in the top functional network, suggesting the involvement of ubiquitination in regulating and/or mediating Notch3 signaling.

Since NEDD4 family of E3 ubiquitin ligases were previously reported to interact with Drosophila Notch receptors and regulate their signaling activity [10], [11], our discovery of WWP2, an E3 ubiquitin ligase belonging to the NEDD4 family, as a strong Notch3-interacting protein, is particularly interesting (Fig. 1A, Table S1). In the following study, we focused on determining the role of WWP2 in controlling Notch3 ubiquitination and receptor signaling.

### Validation of WWP2 as a protein binding partner of N3-ICD

To determine if WWP2 was a bona fide N3-ICD interacting protein, we first tested the interaction between rhN3-ICD-V5 and WWP2-GST proteins using an *in vitro* GST pull-down assay. The results demonstrated that rhN3-ICD-V5 was specifically pulled down by WWP2-GST in a dose-dependent fashion, but was not pulled down by GST-control peptides (Fig. 1B). Since WWP2 is a HECT-domain E3 ligase that regulates ubiquitin-dependent degradation of its substrates, we determined whether WWP2 promoted ubiquitination of Notch3 protein using *in vitro* ubiquitination assays. Recombinant WWP2-FLAG was incubated with rhN3-ICD-V5, ubiquitin (Ub), and the E1 and E2 enzymes. Ubiquitination of Notch3 protein was determined by immunoprecipitation using an anti-V5 antibody followed by Western blotting with an anti-ubiquitin antibody. We found that WWP2 induced heavy ubiquitination of N3-ICD (Fig. 1C). These data demonstrated that N3-ICD directly bound to WWP2, and that Notch3 was an ubiquitination substrate of WWP2.

**Figure 2:**
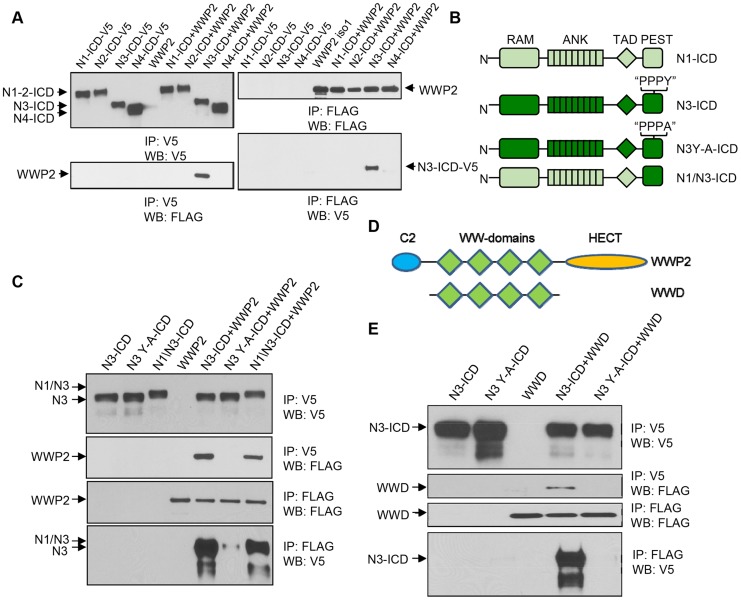
WWP2 binds to N3-ICD but not to N1-ICD, N2-ICD, or N4-ICD. (**A**) Immunoprecipitation (IP) using either anti-FLAG or anti-V5 antibody was carried out using extracts prepared from 293T cells transfected with ICD construct alone or in combination with WWP2 as indicated. The interaction of FLAG-tagged WWP2 with the different V5-tagged ICD was evaluated by western blot (WB) with the respective antibody. Only N3-ICD was co-immunoprecipitated with WWP2. (**B**) Schematic representation of N1-ICD and N3-ICD, along with Y-A mutant of N3-ICD and N1/N3-ICD chimera. (**C**) Immunoprecipitation using either anti-FLAG or anti-V5 beads was carried out using extracts prepared from 293T cells transfected with constructs alone or in combination as indicated. The interaction of FLAG-tagged WWP2 with the different V5-tagged ICD was evaluated by western blot (WB) with their respective antibodies. (**D**) Schematic representation of WWP2 and a construct coding for the four WW domains (WWD). (**E**) Immunoprecipitation using either anti-FLAG or anti-V5 beads was carried out using extracts prepared from 293T cells that were transfected with constructs alone or in combination as indicated. The interaction of FLAG-tagged WWD with the different V5-tagged N-ICDs was evaluated by western blot (WB) with their respective antibodies. Result shows that interaction between N3-ICD and WWP2 is mediated by the WW domain.

To test whether WWP2 specifically bound to N3-ICD but not to other Notch receptors, we performed co-immunoprecipitation assays in HEK293T cells transiently transfected with the WWP2 expression construct along with construct carrying intracellular cytoplasmic domains (NICD) of each of the four human Notch receptors (Notch1–4). We observed that among four NICDs, WWP2 only co-immunoprecipitated with N3-ICD (Fig. 2A). WWP2 belongs to the “WW” domain-containing protein family, which is known to interact with their substrates through the “PPxY” motif [11]. Sequence analysis of the human Notch receptors has shown that the “PPxY” motif is present in the PEST-domain of N3-ICD (presented as PPPY) but is not present in other human Notch receptors [12]. To determine whether the PPPY motif of the N3-ICD was indeed required for the binding between WWP2 and N3-ICD, we generated an N3-ICD PPPA mutant (N3-ICD Y-A) by substituting tyrosine in this motif with alanine. In addition, we created a Notch1-Notch3 chimera by replacing Notch1 TAD and PEST domains with the PEST domain of Notch3 (Fig. 2B). Co-immunoprecipitation experiments were performed in HEK293T cells transiently co-transfected with each mutant construct and WWP2. The results demonstrated that a single point mutation in the PPPY motif of N3-ICD abolished its interaction with WWP2 (Fig. 2C). Furthermore, although N1-ICD cannot interact with WWP2, fusion of the N3-ICD PEST domain with the N1-ICD endowed the newly generated chimeric protein to interact with WWP2 (Fig. 2C). These results indicated that the C-terminal PEST-domain of Notch3 conferred the ability to interact with WWP2.

Reciprocally, we determined if the WW domain of WWP2, which was known to mediate protein-protein interaction, was responsible for interacting with N3-ICD. We created a truncation construct, WWD, which contains the four WW domains of WWP2 (Fig. 2D), and demonstrated that WWD was co-immunoprecipitated with the wild-type N3-ICD but not with the N3-ICD Y-A mutant (Fig. 2E). These experiments confirmed that the PPPY motif in the N3-ICD PEST domain mediated direct interaction with WWP2 via its WW domains.

### Secretase-cleaved Notch3 fragments are ubiquitinated by WWP2

**Figure 3:**
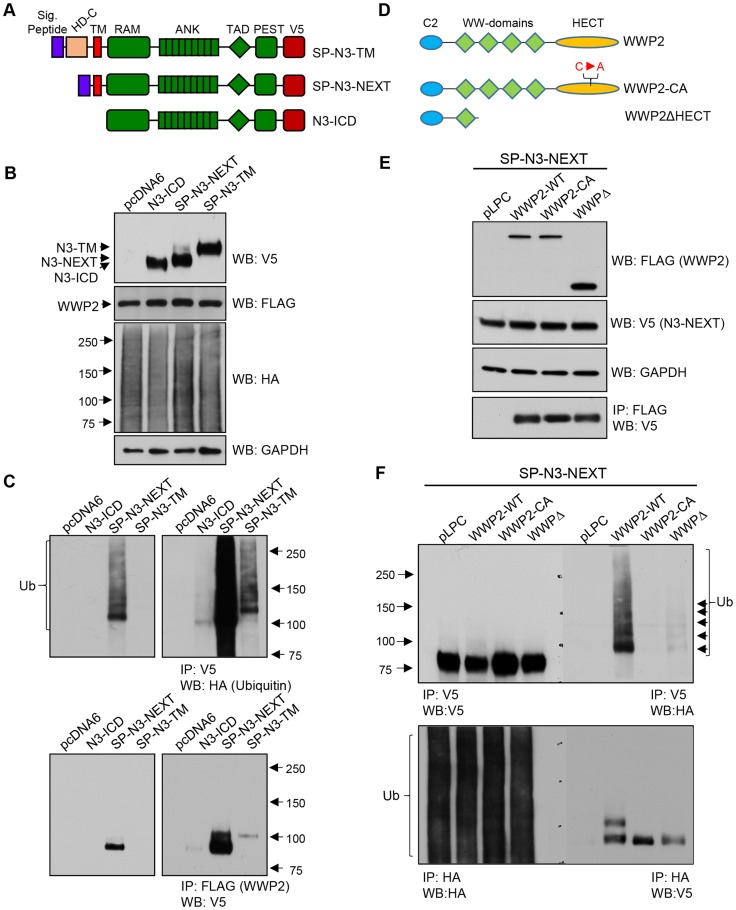
WWP2 ubiquitinates Notch3 receptor and regulates its activity. (**A**) Schematic representation of N3-TM, N3-NEXT, and N3-ICD constructs. Signal peptide sequences were inserted into N3-TM and N3-NEXT constructs. To facilitate detection, all Notch3 variant constructs contained a V5 epitope tag at the C-terminus. (**B**) 293T cells were transfected with HA-tagged ubiquitin together WWP2 and different Notch3 variant constructs: N3-TM, N3-NEXT, or N3-ICD. To determine the expression of different constructs, Western blot analysis was performed with anti-V5, anti-FLAG, or anti-HA, antibody using input lysates. Equal loading was determined with an anti-GAPDH antibody. (**C**) To detect ubiquitinated Notch3, immunoprecipitation (IP) was performed on the cell lysates using anti-V5 beads, and western blot (WB) was performed with an anti-HA antibody. To detect the interaction between Notch3 variant and WWP2, IP was performed by using anti-Flag agarose, and western blot was performed using anti-V5 antibody. Left panel: short exposure; right panel: longer exposure. (**D**) Schematic representation of wild type WWP2, a catalytically inactive C838A mutant of WWP2 (WWP2-CA), and a deletion construct of WWP2 lacking the HECT ubiquitination domain and three WW domains (WWP2ΔHECT). All of the WWP2 expression constructs contained a flag epitope tag at the C-terminal end. (**E**) 293 cells were transfected with N3-NEXT, ubiquitin-HA, and pLPC control plasmid, WWP2, WWP2-CA, or WWP2ΔHECT. To determine expression of the different constructs, western blot analysis of lysates was performed with anti-FLAG or anti-V5 antibody (input lysate). (**F**) To detect ubiquitinated N3-NEXT, immunoprecipitation (IP) was performed on the cell lysates using the anti-V5 beads, and western blot (WB) was performed with an anti-HA antibody. Reciprocal co-IP was performed by using anti-HA agarose beads for pull-down and using the anti-V5 antibody for western blot.

Notch3 receptor is activated through two juxta-membrane protease cleavages: upon ligand stimulation, the alteration in configuration of membrane tethered Notch3 fragment (N3-TM) triggers the first cleavage by α-secretase of the ADMA family to generate N3-NEXT, which contains N3-ICD plus the transmembrane domain (Fig. 3A). Membrane-tethered N3-NEXT is then cleaved by γ-secretase within the transmembrane region to release the soluble intracellular fragment, N3-ICD. N3-ICD quickly translocates into the nucleus and regulates transcription of target genes through its interaction with co-factors including RBPJ. Although our protein microarray screen which used recombinant protein in a cell-free system suggests that WWP2 directly interacts with the final secretase cleavage product of Notch3, N3-ICD, at the cellular level, WWP2 is likely to interact with and ubiquitinate intermediate Notch3 fragments including N3-TM and N3-NEXT. To assess this possibility, we generated N3-TM-V5 and N3-NEXT-V5 expressing constructs and inserted a signal peptide sequence to the N-terminus of these constructs to ensure correct protein topology of these Notch3 fragments (Fig. 3A). *In vivo* ubiquitination status of these fragments was examined by performing co-transfection with flag-tagged WWP2 along with HA-tagged ubiquitin plasmids in 293T cells. The expression of each construct was confirmed by western blot, and the results demonstrated that similar amounts of Notch3 fragments were present in each experimental group (Fig. 3B). WWP2 and ubiquitin were also expressed at comparable levels (Fig. 3B). The level of ubiquitination of Notch3 fragments was measured by reciprocal immunoprecipitation with HA and V5 antibodies. The results demonstrated that all tested Notch3 fragments were ubiquitinated in cells co-transfected with WWP2; however, the ubiquitination level of N3-NEXT was most apparent (Top, Fig. 3C and Fig. S1A). There was a prominent band representing mono-ubiquitinated Notch3 protein and a weak high molecular weight smear corresponding to poly-ubiquitinated products. To test the possibility that the differential ubiquitination levels among the Notch3 fragments was due to their ability to encounter and interact with WWP2 *in vivo*, we performed co-immunoprecipitation experiments in 293T cells following co-transfection of the V5-tagged Notch3 fragment and flag-tagged WWP2. The results demonstrated that although protein expression levels were comparable among the three Notch3 fragments, N3-NEXT protein was more abundantly bound to WWP2 than N3-TM and N3-ICD, reflected by an intense protein pull-down band (Bottom, Fig. 3C). Reciprocal co-immunoprecipitation assays also confirmed the above finding (Fig. S1B).

To determine specificity of Notch3 ubiquitination by WWP2, we generated catalytically inactive WWP2 mutant constructs including C838A and WWP2ΔHECT (Fig. 3D). Flag-tagged wildtype or mutant WWP2 construct was co-transfected with N3-NEXT-V5 and HA-tagged ubiquitin into 293 cells. The expression of each construct was verified by western blot which demonstrated comparable levels of expression in different constructs (Fig. 3E). Co-immunoprecipitation showed a similar binding between N3-NEXT fragment and WWP2 proteins: wildtype WWP2, WWP2ΔHECT mutant and WWP2CA mutant (bottom panel Fig. 3E). *In vivo* ubiquitination was determined by reciprocal co-immunoprecipitation assays using V5 and HA antibodies. The results showed that N3-NEXT was readily mono-ubiquitinated by wild-type WWP2 but not by the catalytically inactive WWP2 mutants (Fig. 3F).

**Figure 4:**
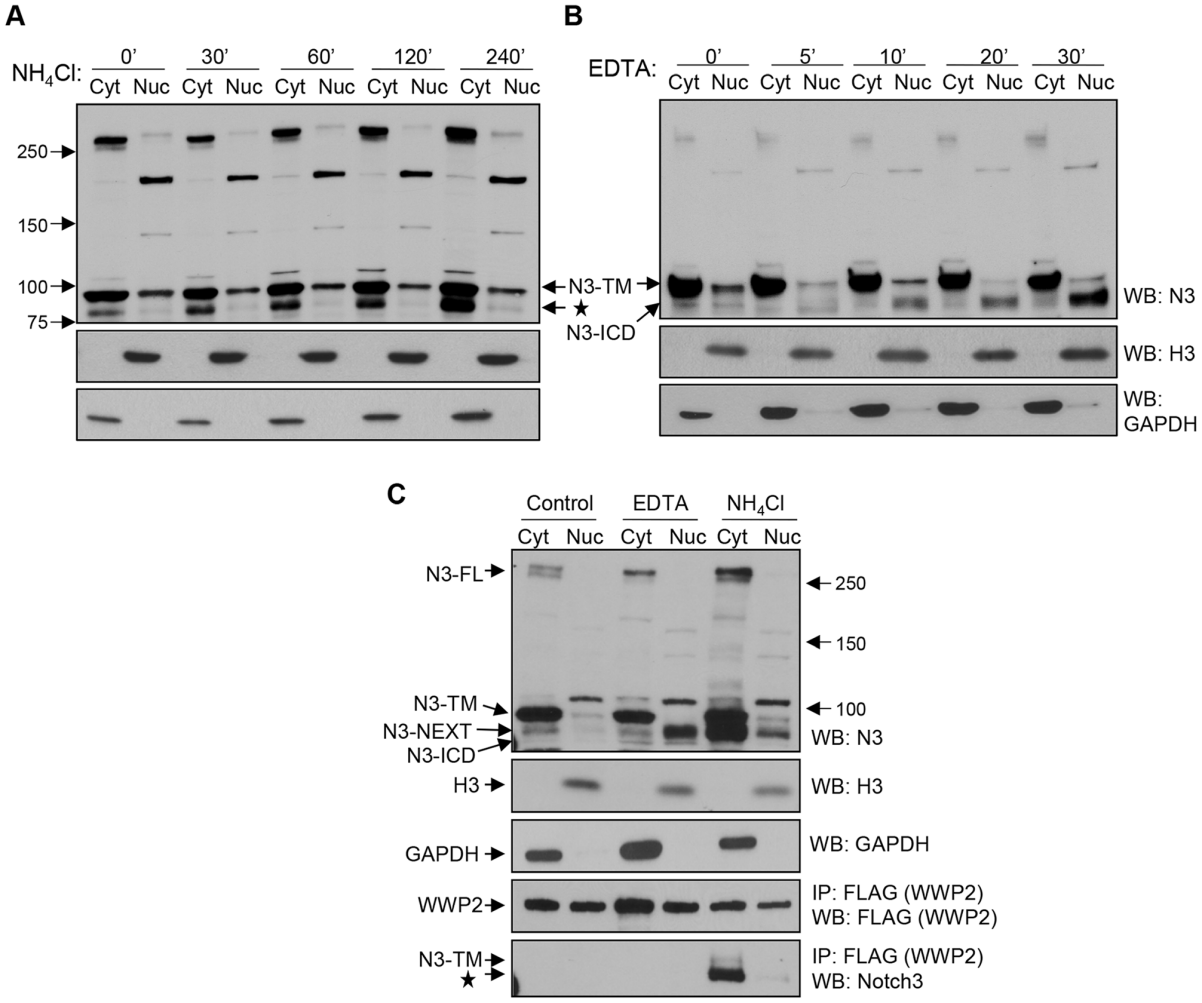
WWP2 interacts with endogenous Notch3 in cancer cells. (**A**) MCF7 cells were incubated with 25 mM NH4Cl for 0, 30, 60, 120, and 240 minutes. Cells were lysed and the lysates separated into nuclear (N) and cytosol/membrane (C) fractions. Western blot analysis was performed with an anti-Notch3 antibody. Equal loading was determined with an anti-GAPDH antibody for the cytosol/membrane fraction and with an anti-histone 3 antibody for the nuclear fraction. The star represents the N3-NEXT bands. (**B**) MCF7 cells were incubated with 2.5 mM EDTA for different times as indicated to induce Notch-ICD generation. Cells were lysed and fractionated into nuclear (N) and cytosol/membrane (C) fractions. Western blot analysis was performed with an anti-Notch3 antibody. (**C**) Ovarian cancer cells were first transfected with the flag tagged WWP2 expressing plasmids. The cells were then treated with 2.5 mM EDTA for 20 minutes or with 25 mM NH4Cl for 240 minutes, and then fractionated into nuclear and cytosol/membrane fractions. Immunoprecipitation (IP) was performed with anti-flag agarose beads for pull-down and a rabbit anti-Notch3 antibody for western blot. Expression of GAPDH and histones were used for demonstrating the purity of nuclear and cytosol/membrane fractions, respectively.

A previous study demonstrated that Notch3 protein degradation occurs in the endosome/lysosome compartments [13]; therefore, we determined whether Notch3 cleavage fragments accumulated in OVCAR3 and MCF7 cancer cells in the presence of a lysosomal inhibitor. Representative figures were shown in Fig. 4A, demonstrating that we were able to confirm this prior finding. Furthermore, we demonstrated that after lysosomal blockage by NH_4_Cl, the faster migrating band corresponding to post-secretase cleavage products of Notch3 accumulated in the membrane/cytosol fraction but not in the nuclear fraction (Fig. 4A). As a control, we used EDTA to trigger Notch3 cleavages and generate soluble N3-ICD. In this case, the released N3-ICD was detected exclusively in the nuclear fraction (Fig. 4B). To determine if WWP2 interacted with endogenous Notch3 fragments, we ectopically expressed WWP2-FLAG in OVCAR3 cells because WWP2 expression is relatively low in most tested ovarian cancer cell lines. Then we performed co-immunoprecipitation experiments using an anti-FLAG antibody and an antibody recognizing endogenous Notch3. The cells were assayed in the presence or absence of the lysosomal blocker, NH_4_Cl. Inhibition of lysosomal degradation increased detectable association of WWP2 with the secretase cleaved Notch3 fragments in the cytoplasmic fraction (Fig. 4C). This is likely attributable to increased levels of secretase cleaved Notch3 fragments after lysosomal blockage. We note that only the faster migrating band corresponding to secretase cleaved products of Notch3 was co-immunoprecipitated with WWP2, whereas the pre-cleaved form of Notch3, TM, was not. We also performed EDTA treatment and subcellular fractionation to test if endogenous N3-ICD interacted with WWP2. Co-immunoprecipitation experiments demonstrated that although abundant N3-ICD fragments were detected in the nuclear fraction, and WWP2 was also present in the nucleus, there was no detectable interaction between them under this condition (Fig 4C).

We also applied immunofluorescence staining to visualize cellular localization of WWP2 and Notch3 after treatment of a lysosomal blocker, NH_4_Cl. As shown in Fig. S2A, upon NH_4_Cl treatment, both WWP2 and Notch3 protein fragments co-localized predominantly in the cytoplasm. In contrast, when cells were treated by EDTA (Fig. S2B), Notch3 predominantly localized to the nucleus, most likely because of the induced cleavage by secretases to release N3-ICD (Western blot shown in Fig. 4B). However, under this condition, WWP2 was primarily localized to the cytoplasm, so there is minimal co-localization between WWP2 and N3-ICD. Therefore, the immunofluorescence result was consistent with the above co-immunoprecipitation study.

### Deletion of WWP2 locus in ovarian carcinomas

**Figure 5:**
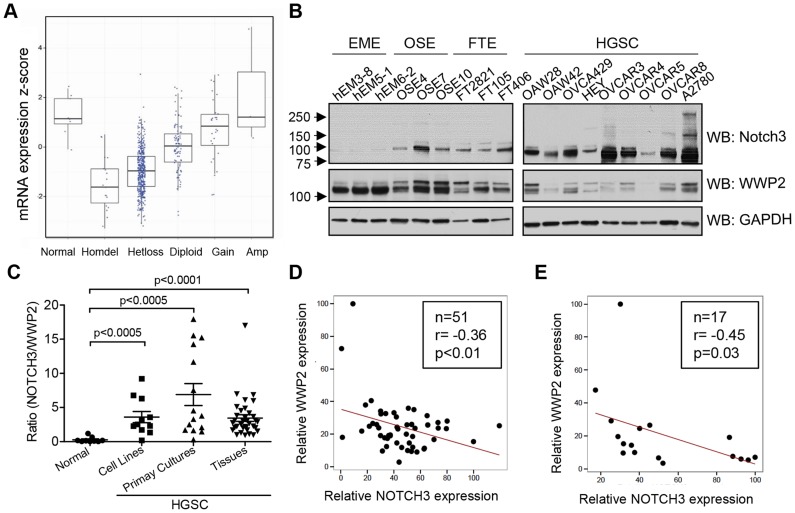
WWP2 is down-regulated in ovarian carcinomas. (**A**) Analysis of DNA copy number at the *WWP2* locus and WWP2 transcript expression levels in TCGA's ovarian HGSC dataset. Among 554 HGSCs, heterozygous allele loss (hetloss) was observed in 410 tumors and homozygous deletion in 18 cases (homdel). (**B**) WWP2 and Notch3 protein expression in ovarian cancer cell lines (HGSC) and in non-transformed epithelial cell cultures established from human gynecologic organs. These include endometrial epithelial cells (EME), ovarian surface epithelial cells (OSE), and fallopian tube epithelial cells (FTE). GAPDH is included as the loading control. (**C**) Band intensities of WWP2 and Notch3 from immunoblotting were individually quantified and normalized to GAPDH for each sample. The ratio of Notch3 and WWP2 protein expression was determined. The ratio is higher in ovarian cancer groups (HGSC) than in normal epithelial cell group. Two-tailed Man-Whitney U test was performed to determine the level of significance. (**D and E**) Correlations of protein expression between WWP2 and Notch3 in 51 ovarian cancer tissue samples (D) and in 17 primary cultures of ovarian cancer cells (E). There is an inverse correlation between Notch3 expression and WWP2 expression with a Pearson's correlation coefficient of −0.36 and p<0.01 for tumor tissue samples and a Pearson's correlation coefficient of −0.45 and p<0.05 for primary cultures.

We have previously demonstrated *Notch3* gene amplification and over-expression in ovarian high-grade serous carcinoma (HGSC) [4]. If WWP2 is a negative regulator of Notch3, its expression is expected to be down-regulated in ovarian cancer as compared to normal tissues. To test this hypothesis, we examined the copy number alteration and expression pattern of WWP2 in ovarian HGSCs using a large summarized TCGA dataset [14]. In the 554 ovarian HGSCs that have available copy number data, the majority (77.3%) of HGSCs harbored deletions at the *WWP2* locus, including 410 hemizygous and 18 homozygous deletions (Fig. 5A). In addition, based on a GISTIC copy number analysis in ovarian HGSC samples, the *WWP2* locus is in a significantly deleted region (q-value = 10^−13^, Fig. S3) [15]. To define the minimal region of homozygous deletion- a strategy commonly used to identify potential deleted tumor suppressor gene, we aligned the homozygously deleted regions of the 18 HGSC samples and found that the region spans from 69,942,965 bp to 69,976,479 bp on chromosome 16 (hg19), which only encompasses *WWP2* gene. To determine if loss of WWP2 DNA copy number affects WWP2 transcription, we determined the relationship between the gDNA and mRNA copy numbers of WWP2 using all HGSCs available in the TCGA. The result demonstrated a significant positive correlation between WWP2 gDNA copy number and WWP2 mRNA expression levels in HGSCs (Fig. S4, Pearson's r = 0.6863, p<0.0001).

As protein ubiquitination plays a major role in regulating the steady state level of protein expression, we determined the relationship between Notch3 protein expression and WWP2 expression using semi-quantitative Western blot analysis. Immunoblotting was performed on ovarian cancer cell lines, ovarian HGSC tissues, primary cultures of ovarian carcinomas, and non-transformed epithelial cells derived from female reproductive organs (endometrium, fallopian tube and ovarian surface), and WWP2 and Notch3 protein expression levels were quantified using a densitometer. As shown in Fig. 5B, an increase in Notch3 expression level and a decrease in WWP2 level were observed in ovarian cancer cell lines as compared to normal non-transformed epithelial cells. The ratio of Notch3 expression level to WWP2 expression level in all samples was shown in Fig. 5C. There was a significantly higher Notch3 to WWP2 protein expression ratio than the normal epithelial cells in ovarian cancer tissues, primary cultures of ovarian cancer cells, and ovarian cancer cell lines. When expression levels of Notch3 was plotted against WWP2 expression levels in ovarian cancer tissues and primary cultures, an inverse correlation in protein expression levels between WWP2 and Notch3 was observed. The result indicated that ovarian HGSC tissues (Fig. 5D) and primary tumor cell cultures (Fig. 5E) with higher Notch3 expression levels tended to have lower expression levels of WWP2, and vice versa.

### WWP2 expression suppresses cellular proliferation and Notch signaling activity in cancer cells

**Figure 6:**
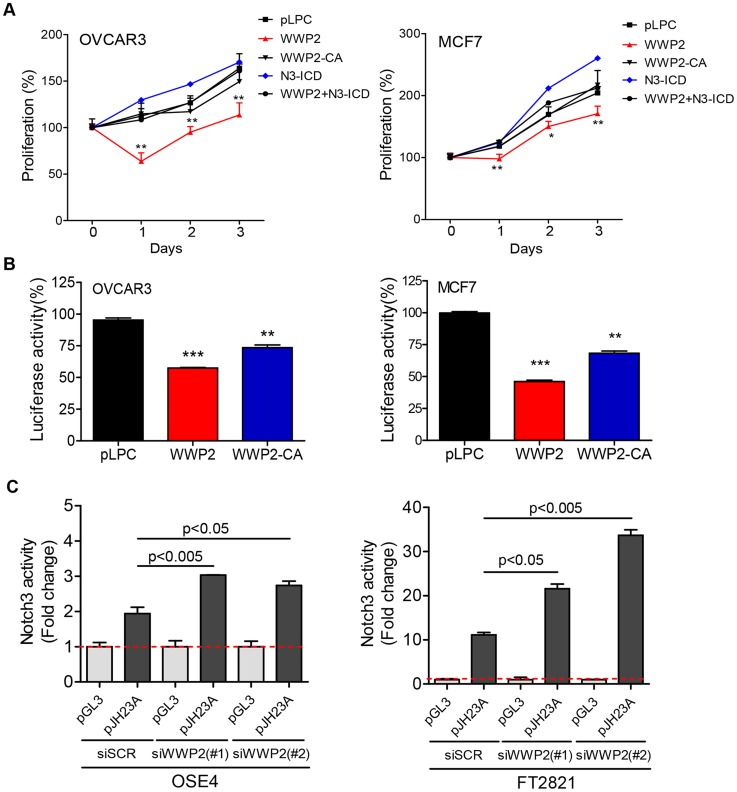
WWP2 regulates Notch3 signaling activity in cancer cells. (**A**) OVCAR3 or MCF7 cancer cells were transfected with control plasmid pLPC, WWP2, catalytically-inactive WWP2 (WWP2-CA), N3-ICD, or WWP2+N3-ICD. Relative cell numbers were measured at different time points using the SYBE Green-based assay. Data are expressed as means ± SD and Student's *t*-test was performed to compare the relative cell number between WWP2+N3-ICD and pLPC (* p<0.05; ** p<0.01). (**B**) Different groups of cells were transfected with the pJH23A (4×wtCBF1Luc) luciferase reporter construct together with pLPC, WWP2, or the catalytically-inactive WWP2 plasmid (WWP2-CA). Each experiment was performed in triplicate. Results are shown as percentage of luciferase activity compared to pLPC transfected controls. Data are expressed as means ± SD. P values were calculated by comparison between either WWP2 or WWP2-CA and the control plasmid pLPC (** p<0.01; *** p<0.001). (**C**) Two untransformed epithelial cell lines were transfected with WWP2 siRNA or scrambled siRNA (siSCR). Notch signaling was measured by co-transfecting the cells with a luciferase reporter plasmid, pJH23A (4×wtRBPJLuc) and the data were normalized to the luciferase activity measured by co-transfection with a control plasmid, pGL3. Data are expressed as means ± SD and statistical significance was assessed by two-tailed Student's *t*-test.

To determine whether reconstitution of WWP2 expression in cancer cells resulted in reducing Notch3 signaling activity and subsequently cellular proliferation, we performed proliferation assays in OVCAR3 and MCF7 cells transduced with pLPC control plasmid, N3-ICD, WWP2, or catalytically inactive mutant WWP2-C838A (WWP2-CA). Proliferation significantly decreased in both cell lines when WWP2 was ectopically expressed as compared to cells transfected with the control plasmid or cells expressing the WWP2-CA (Fig. 6A). We also determined if N3-ICD could reverse the anti-proliferative effect imposed by WWP2 by co-transfecting cells with N3-ICD and WWP2 expression plasmids. Our data demonstrated that N3-ICD counteracted the growth-inhibitory effect of WWP2, so the growth curve of cells co-transfected with N3-ICD and WWP2 was comparable to the curve of cells transfected with control plasmid (Fig. 6A). Using a Notch signaling reporter assay, we also observed reduced endogenous Notch signaling activity when WWP2 was expressed in MCF7 and OVCAR3 cells (Fig. 6B). Expression of the enzymatically inactive mutant WWP2-C838A in MCF7 and OVCAR3 cells also reduced Notch signaling activity, however to a lesser extent. To confirm the phenotypes observed in cancer cell lines, we performed experiments in primary ovarian tumor cultures. WWP2 cDNA or control vector was co-transfected with a Notch signaling reporter, pJH23A, into primary cell cultures and Notch signaling activity of WWP2 cDNA -transfected group was measured and the data was normalized to the data obtained from the control vector-transfected group. The normalized data were plotted against Notch3 expression levels measured in the same tumor samples (Fig. S5A). The result demonstrated that ectopic expression of WWP2 potently suppressed Notch signaling in cells with high levels of Notch3 (reflecting by greater reduction of Notch signaling activity in the Notch3-high cells) (Fig. S5B).

Next, we employed a loss-off-function approach to assess the contribution of WWP2 on Notch signaling. Expression of WWP2 in untransformed cell lines including OSE4 (an ovarian surface epithelial cell line) and FT2821 (a fallopian tube epithelial cell line) was down-regulated by using siRNAs. The knockdown efficiency of two different WWP2 targeting siRNAs was validated by Western blot (Fig. S6). To detect the effect on Notch signaling, cells were co-transfected with a Notch signaling reporter, pJH23A, or with a control vector pGL3. The data demonstrated that downregulation of WWP2 by siRNAs significantly increased Notch signaling activity in both untransformed cell lines (Fig. 6C).

**Figure 7:**
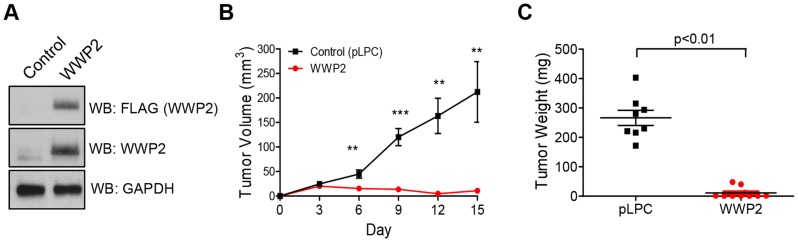
WWP2 suppresses tumor development of ovarian cancer cells. (A) Tumorigenic SKOV3-CR cells were transfected with either the plasmid encoding WWP2 cDNA or the control plasmid, pLPC. The expression of WWP2 was determined by Western blot. (B) Cells were injected into the right flanks of athymic *nu/nu* mice. The tumor size was measured by a caliper every three days. Two-tailed Student's *t*-test was used to determine the significance level. ** p<0.01; *** p<0.001 (C) Tumor weight was measured at the end point. Two-tailed Student's *t*-test was employed to determine the significance level.

To determine whether WWP2 expression affects tumor development, we ectopically expressed WWP2 in SKOV3-CR cells which were pre-established to develop carboplatin resistance (CR) phenotype and expressed abundant Notch3. SKOV3-CR cells transfected with the empty vector, pLPC, served as a control. The presence or absence of ectopic WWP2 expression in these transfected cells was validated by Western blot (Fig. 7A). A total of 3×10^6^ cells were injected subcutaneously into the athymic *nu/nu* mice and tumor growth was monitored every three days. We observed that WWP2 expression led to a significant reduction in tumor formation and in fact as compared to the control group, tumors in the WWP2 expressing group were hardly detectable (Fig. 7B). Tumor weight measured at the endpoint of the study was also significantly lower in the WWP2-expressing group than in the control group (Fig. 7C).

Inhibition of Notch3 by siRNA was previously shown to lead to cell cycle arrest in the G2/M phase in OVCAR3 cells [8] and to cause G0/G1 arrest in MCF7 cells [16]. To determine if WWP2 expression leads to similar cell cycle arrests, WWP2 expression plasmid or pLPC control plasmid was transfected into OVCAR3 and MCF7 cells. Results of cell cycle analysis on these cells were consistent with results from Notch3 knockdown. Overexpression of WWP2 in OVCAR3 cells led to a significantly increased population of cells within the G2/M phase and a simultaneous loss of cells within the G0/G1 population (Fig. S7). Overexpression of WWP2 in MCF7 cells led to increased cell numbers within the G0/G1 phase and reduced numbers within G2/M, demonstrating the functionally diverse outcomes in different cellular backgrounds.

**Figure 8:**
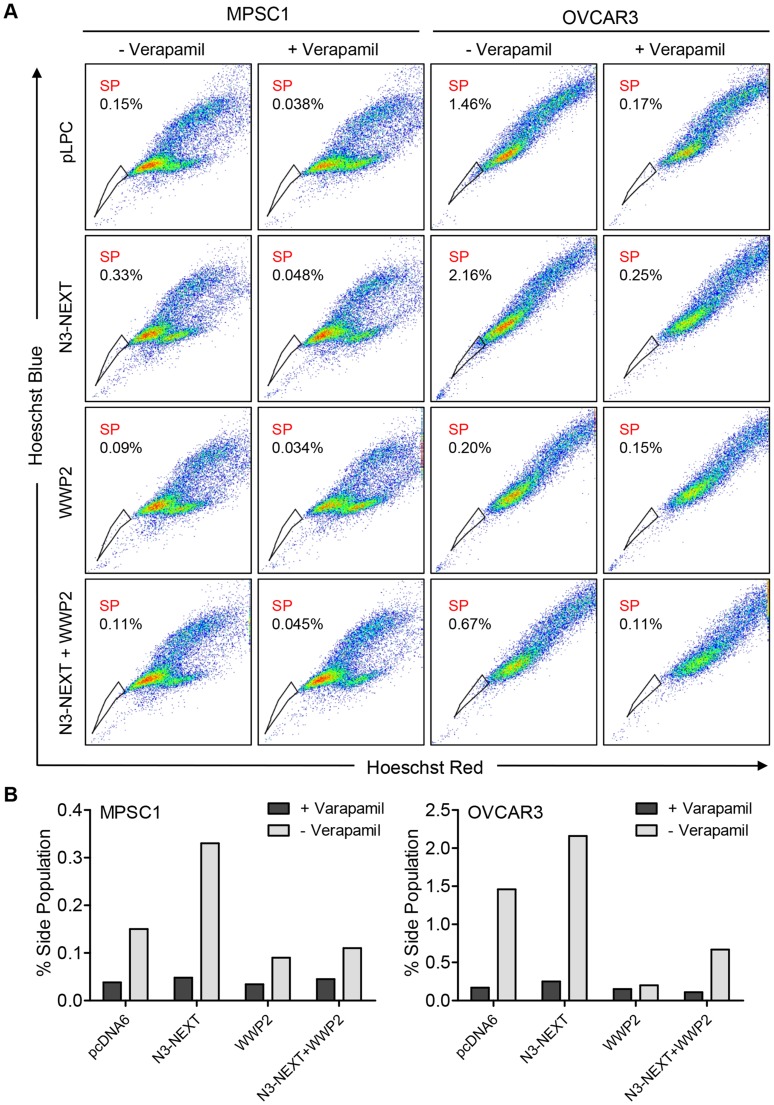
WWP2 counteracts Notch3-induced increase in CSC population. (A) N3-NEXT overexpression (second row) increased frequencies of verapamil-sensitive SP cell in MPSC1 and OVCAR3 cells compared with pLPC vector-transfected control (first row). Ectopic expression of WWP2 reduced SP fraction in both cell lines (third row). Co-expression of WWP2 in N3-NEXT-expressing cells reduced SP fraction (fourth row) as compared to cells with only N3-NEXT overexpression (second row). (B) Bar charts represent % of SP in each experimental condition.

Previous studies by our group and others have shown that Notch3 upregulation is related to the recurrence of ovarian cancer and is associated with a poor prognosis [7]. In addition, Notch 3 overexpression increases the proportion of cancer stem cell-like cells (CSCs) and resistance to platinum-based therapy [7], [17]. To determine whether WWP2 can counteract these Notch3-mediated phenotypes, we performed flow cytometry and Hoechst 33342 dye efflux assay to measured side population which is enriched with CSCs. Ovarian cancer cells were transfected with empty vector, N3-NEXT, WWP2, or N3-NEXT+WWP2. The flow cytometry results demonstrated that overexpression of Notch3 increased the side population fraction but the percentage of cells in this population was reduced when WWP2 was also expressed. Thus, the presence of WWP2 counteracts Notch effect on promoting the CSC phenotype (Fig. 8). Similarly, expression of WWP2 decreased the Notch3-induced platinum resistance as evidenced by analyzing cell viability in OVCAR3 cells cultured with carboplatin (Fig. S8).

## Discussion

It has been established that Notch3 plays a fundamental role in a variety of cellular functions including cellular differentiation, organ development, and cancer pathogenesis. However, the mechanisms that regulate and mediate Notch3 function remain largely unknown. To address the issue, this study aimed to identify Notch3 interacting proteins using an unbiased, comprehensive protein chip-based approach. In this screen, the canonical Notch receptor co-factor, RBPJ, exhibited high binding affinity to Notch3 receptor, indicating the efficiency of the approach. In addition to RBPJ, a list of potential Notch3 receptor-interacting proteins was identified. These proteins are involved in functional networks including protein ubiquitination, cell signaling, and transcriptional regulation. Their identification provides new insight into the Notch3 signaling network. We chose one of the Notch3 interacting proteins, WWP2, in the ubiquitination pathway, for further investigation.

Protein ubiquitination is a posttranslational modification of a specific protein that leads to a unique fate through protein trafficking. For example, mono-ubiquitination of membrane proteins triggers their endocytosis and targeting to endosomes/lysosomes [18]; whereas K48-linked polyubiquitination is a signal for targeting cytosol proteins to the proteasome for degradation [18]. Genetic studies in Drosophila have demonstrated that the Nedd4 family of ubiquitin ligases, including Suppressor of Deltex and Nedd4, negatively regulate Notch receptor signaling [10]. In mammals, a homolog of the Nedd4 family, Itch/AIP4, was found to ubiquitinate membrane-bound Notch1 receptor (N1-TM) prior to its activation, and to target its endocytosis and endosomal trafficking [12], [19]. More recently, it has been demonstrated that in contrast to Drosophila Nedd4, mammalian Itch does not interact directly with Notch1 receptor. Its ability to negatively regulate Notch signaling is thought to be through interaction with the adaptor protein, Numb [20]. This result is not surprising because unlike Drosophila Notch or mammalian Notch3, mammalian Notch1 lacks the PPxY motif in the C-terminal region which is critical for direct interaction with E3 ubiquitin ligases in the Nedd4 family [12]. Compared to the membrane-tethered form, the soluble active form of Notch1, N1-ICD, has been shown to be directly polyubiquitinated by Sel-10 (FBXW7), a constituent of the SCF ubiquitin protein ligase complex, and was subsequently targeted to proteasome for degradation [21].

These prior studies demonstrated that Notch1 activity can be regulated by multiple ubiquitination-mediated mechanisms. Comparatively little is known about regulation of other Notch receptors such as Notch3. In this study, using comprehensive proteome microarray, we have identified WWP2, a NEDD4 family member of E3 ubiquitin ligase, as a negative regulator of Notch3 signaling in ovarian cancer. We have shown that WWP2 directly interacts with and mono-ubiquitinates post-secretase cleaved Notch3 fragments, promoting their sorting to and degradation in lysosomes, thereby suppressing Notch3 signaling activity in cancer cells. Among the four Notch receptors present in mammals, only Notch3 contains the PPxY motif within its N3-ICD PEST domain. The PPxY motif is evolutionary conserved from Drosophila to mammals and is responsible for direct interaction with the WW domain of WWP2. The PPxY motif in Drosophila Notch was shown to be responsible for direct interaction with Drosophila Nedd4 and targeting the receptor for endocytosis and endosomal sorting [11]. In this study, we found that WWP2 promotes a strong mono-ubiquitination pattern of post-α-secretase cleaved, membrane tethered fragments of Notch3, N3-NEXT. This ubiquitination pattern has been suggested to serve as the “seed” for endocytic protein interaction networks. Ubiquitination occurred to a lesser extent in the “resting form” N3-TM or the cytosolic soluble form, N3-ICD. We speculate that the favorable ubiquitination towards N3-NEXT is triggered by juxta-membrane α-secretase cleavage which leads to a conformational alteration in the Notch3 fragments and increases their accessibility to WWP2.

A recent study has described endocytosis of the Notch3 receptor and subsequent lysosomal degradation of both extracellular and intracellular fragments of Notch3 [13]. Accumulation of N3-ECD (extracellular fragments) as well as N3-ICD (intracellular fragments) was observed in the presence of the lysosomal inhibitors but not in the presence of the proteasome inhibitor. In contrast to its effect on Notch3, proteasome inhibitor leads to accumulation of intracellular fragments of Notch1 (N1-ICD). In this study, we have confirmed the above findings in an ovarian cancer cell line, OVCAR3, and in a breast cancer cell line, MCF7, both of which express abundant Notch3. In addition to Notch receptors, OVCAR3 and MCF7 also express high levels of Notch ligands including Jagged1 and Delta4, respectively, which may provide a constitutive cue for Notch3 signal activation. Treatment with NH_4_Cl, which suppresses lysosomal degradation of Notch3 fragments, significantly increased the interaction between WWP2 and post-secretase cleaved fragments of Notch3, suggesting that WWP2 regulates targeting of Notch3 to the endosome/lysosome compartment where the receptor is degraded. Since WWP2 contains a C2 domain primarily found in proteins regulating membrane trafficking and mediating binding to phospholipids, raising a possibility that the primary subcellular interaction site between WWP2 and Notch3 is located at juxta-plasma membrane region.

In addition to demonstrating WWP2 as a regulator for Notch3 trafficking and signaling activity, this study has established a tumor suppressor role of WWP2 in ovarian cancer. We reported that WWP2 is genetically deleted and down-regulated in a significant number of ovarian high-grade serous carcinomas and overexpression of WWP2 suppresses tumor development and causes cell cycle arrest. Furthermore, we demonstrated that WWP2 expression counteracts the Notch3 promoted phenotypes including CSCs and platinum resistance. The negative regulation of WWP2 on Notch signaling activity is more prominent in Notch3-overexpressing cancer cells. This could be because those cancer cells are more dependent on Notch signaling for maintaining cellular survival, promoting CSC population and developing platinum resistance. Since WWP2 targets Notch3 and promotes its degradation, enforced expression of WWP2 is expected to result in a more prominent anti-Notch effect in cells expressing abundant Notch3 (or Notch3-dependent cells) than in cells with low or minimal Notch3 expression (or Notch3-indepdenent cells). The reported negative regulatory function of WWP2 on Notch3 signaling could be exploited for designing new strategies to target Notch3 signaling pathway. For example, reagents upregulating the expression of WWP2 and gene therapy approach by re-introducing WWP2 into cancer cells are worth pursuing for the treatment of cancers that have developed dependence on Notch3 signaling.

## Materials and Methods

### Protein microarray screen

The human proteome microarray was fabricated by spotting 16,368 unique, full-length human recombinant proteins in duplicate along with control proteins including IgG, GST, and histones as previously described [9]. Recombinant human N3-ICD-V5 (Met1663-Ala2321) purified from *E. coli* was previously reported [8]. Protein-binding assays on the human proteome microarrays were performed using protocols reported previously [9]. In brief, the microarray was first blocked with 2% BSA in 1× PBS for 2 hr at room temperature, incubated with purified N3-ICD proteins for 1 hr at room temperature, and washed in TBST, followed by incubation with mouse anti-V5 antibody (Invitrogen) at 1∶5000, and then incubated with 1∶1000 Alexafluor 647 conjugated goat anti-mouse antibody (Invitrogen) for detection. After drying, the protein microarray was scanned using a GenePix 4000B scanner (Molecular Devices, Sunnyvale, CA) and signal intensity was calculated as the ratio of median foreground and median background signals in the Cy5 channel.

To quantify the affinity of N3-ICD to each interacting protein on the microarray, we first calculated the mean and standard deviation of the signal intensity across all spots on the microarray. We obtained a normalized signal intensity score for any given protein spot using ratio of median foreground and median background fluorescence as described [22]. Positive interaction spots found in one unique row pool and one column pool that showed intensity score greater than 2.8 for both duplicate spots of any given protein were flagged for individual analysis. Table S1 lists the N3-ICD-interacting proteins with intensity scores greater than 7.0 that were positive in duplicate spots.

### Cell lines

The cell lines used in this study included ovarian cancer cell lines OVCAR3, MPSC1, and SKOV3-CR, an immortalized ovarian surface epithelial cell line OSE4, an immortalized fallopian tube epithelial cell line FT2821, a breast cancer cell line MCF7, and an embryonic kidney epithelial cell line HEK293. SKOV3-CR was developed by incubating parental SKOV3 with low dosage (10 µM) of carboplatin for 3 months. As a result SKOV3-CR became resistant to carboplatin. OVCAR3, MPSC1, SKOV3-CR, OSE4 and FT2821 were maintained in RPMI1640 supplemented with 5% fetal bovine serum and antibiotics. MCF7 and HEK293 cells were maintained in DMEM supplemented with 5% fetal bovine serum and antibiotics. There was no evidence of mycoplasma contamination based on a PCR assay.

### Co-immunoprecipitation experiments

Expression plasmids were transfected into HEK293, OVCAR3, or MCF7 cells using Lipofectamine 2000 (Invitrogen). Cells were harvested 24 hr post-transfection for co-immunoprecipitation experiments. Cells were lysed in lysis buffer (50 mM Tris, pH 8.0, 150 mM NaCl, 1% NP40 supplemented with protease inhibitor cocktail (Thermo Scientific)). Lysates were then incubated with either anti-V5 agarose (Sigma, St. Louis, MO), or anti-FLAG M2 affinity gel (Sigma, St. Louis, MO). After five washes (3 times with lysis buffer and 2 times with TBS) precipitates were resuspended in Laemmli sample buffer containing 5% β-mercaptoethanol.

### Western blot analysis

Samples were separated by 6% or 4–15% SDS-PAGE (Bio-Rad), and were transferred onto a PVDF membrane (Amersham) using a semi-dry transfer apparatus (Bio-Rad). The membrane was blocked with 5% non-fat dry milk (Bio-Rad) or 3% BSA (Sigma) in TBST (20 mM Tris-HCl, 0.5 M NaCl, 0.1% Tween 20) and incubated with a primary antibody, followed by washes with TBST. Subsequently, the membrane was incubated with horseradish peroxidase-conjugated secondary antibody (Jackson Laboratories, West Grove, PA) and detected with ECL developing solution (Thermo Scientific). Western blot was performed using antibodies as indicated.

To correlate protein expression of WWP2 and Notch3, we analyzed 51 ovarian HGSC tissues using Western blot analysis with specific antibodies. Normal counterparts of ovarian cancers including primary cultures of human endometrial epithelial cells (EMs) and fallopian tube epithelial cells (FTs)as well as ovarian surface epithelial cell lines (OSE4, OSE7, and OSE10) were included as controls. Cancer tissues and cells were lysed with lysis buffer (50 mM Tris, pH 8.0, 150 mM NaCl, 1% NP40), supplemented with protease inhibitor cocktail (Thermo Scientific). Cell lysates were subsequently subjected to Western blot analysis. The intensities of WWP2, Notch3, and GAPDH were measured using the ChemiDoc XRS and Image Lab software (Bio-Rad). The intensity of WWP2 or Notch3 was then normalized to the intensity of GAPDH and relative expression value was calculated using the following formula: Δ(Int_A or B_/Int_GAPDH_)/highest(Int_A or B_/Int_GAPDH_)×100.

### Plasmid constructs and molecular cloning

The human WWP2 full length clone (ID:5588092) was purchased from Open Biosystems in pCMV-sport6, PCR amplified with Pfu Ultra II polymerase (Agilent) according to the manufacturer's protocol and cloned with EcoRI and XhoI into the pLPC-N-FLAG plasmid (purchased from Addgene). A construct coding for the four WW domains (WWD) was PCR amplified from full length WWP2 (Ala 274- Gly 478 (820 bp–1434 bp)) and cloned with EcoRI and XhoI into the pLPC-N-FLAG plasmid. WWP2 ΔHECT, coding for the CA2 domain and the first WW-repeat was purchased from Invitrogen (ultimate ORF clone IOH 4735) and cloned into pLPC-N-FLAG plasmid with the Gateway LR clonase II enzyme mix (Invitrogen). WWP2 C838A was generated by site directed mutagenesis (TG 2512–2513>GC (Cys>Ala)) using a kit from Agilent according to the manufacturer's protocol.

The N3-ICD construct has been described previously [7]. Notch1-ICD (5260–7665 (Val 1754-Lys 2555)) and Notch2-ICD (5260–7665 (Val 1754-Lys 2555)) were PCR amplified with Pfu Ultra II polymerase from cDNA transcribed from total RNA derived from SKOV3 and MCF7 cells, respectively, and cloned with NotI and BamHI into pCDNA6-A V5 HIS (Invitrogen). The Notch4 full length ORF clone 9021650 was purchased from Open Biosystems. Notch4-ICD (4399–6009 (Val 1467-Lys 2003)) was PCR amplified and cloned into pCDNA6-A V5 HIS. A Notch1/Notch3 chimera was developed by amplifying the RAM domain and ANK repeats of Notch1 (5260 bp–6342 bp) and the C-terminal PEST domain of Notch3 (6076 bp–6963 bp) with overlapping primers followed by a fusion PCR using the Notch1 forward primer and the Notch3 reverse primer. The fusion fragment was then cloned into the pCDAN-6-V5 expression plasmid with NotI and BamHI.

The human Notch3 full length clone was a kind gift from Dr. Michael Wang (University of Michigan). PCR amplification of N3-NEXT and N3-TM were performed with Pfu Ultra II polymerase (Agilent) and cloned into the pcDNA6-V5 plasmid (Invitrogen) with the addition of a V5 tag in the constructs. To add signal peptide to these constructs, PCR amplified N3-NEXT and N3-TM products were inserted in frame into the plasmid, pSF-CMV-NH2-InsulinSP1 (purchased from Oxford Genetics, UK). The final fusion products included the V5 tag from pcDNA6 and signal peptide from insulin. All constructs were confirmed by sequencing (Macrogen USA). The sequence of cloning primers is available upon request.

### *In vivo* ubiquitination

HEK293T cells were transfected with various combinations of Notch3 and WWP2 plasmids together with HA-tagged ubiquitin. Cells were harvested 24 hr after transfection and lysed in lysis buffer (50 mM Tris, 150 mM NaCl, 1% NP-40 with complete inhibitor (Roche)). Lysates were subjected to immunoprecipitation with anti-HA, anti-FLAG, or anti-V5 beads. Ubiquitination was analyzed by immunoblotting of the anti-HA precipitates with anti-V5 antibody (Bethyl).

### *In vitro* ubiquitination

The reactions were carried out at 37°C for 45 min in 25 µl of ubiquitination reaction buffer (40 mM Tris-HCl at pH 7.6, 2 mM DTT, 5 mM MgCl_2_, 0.1 M NaCl, 2 mM ATP) containing the following components: 250 µM ubiquitin, 100 nM E1 (UBE1), 200 nM UbcH5b (all from Boston Biochem). Purified FLAG-WWP2 was added to the reaction. rhN3 (0.5 µg) was used as a substrate in the reactions as indicated. After the ubiquitination reaction, lysis buffer was added, and samples were incubated with V5-agarose beads (Sigma) for 2 hr. The beads were washed five times with lysis buffer and TBS and boiled in SDS–PAGE loading buffer. Ubiquitination of rhN3 was monitored by Western blotting with anti-ubiquitin antibody (Cell Signaling). An aliquot of each reaction was used directly for Western blotting to demonstrate equal amounts of substrate and/or E3 ligase in the samples as indicated.

### GST pulldown

Bacterial-expressed GST–WWP2 or control GST bound to glutathione–Sepharose beads (Amersham) was incubated with rhN3-ICD for 1 hr at 4°C. The washed complexes were eluted by boiling in SDS sample buffer and separated by SDS–PAGE, and the interactions were analyzed by Western blotting. An aliquot of flow through was used to confirm depletion of rhN3 in presence of WWP2.

### Cellular fractionation

Separation of cell nuclei and membrane/cytosol was performed as previously described (Lee and Green, 1990) with the following modifications: Cells were scraped off and washed twice with cold PBS and once with 20 ml of buffer A (10 mM Tris-HCl, pH 7.4, 8.3 mM KCl, 1.5 mM MgSO_4_, 1.3 mM NaCl). Cells were then swollen on ice for 30 min in buffer A. Nuclei/membranes and cytosol were separated by passing the suspension eight times through a 23-gauge needle followed by 20 rounds through a glass-glass homogenizer. Nuclei and membranes were pelleted by centrifugation at 3000 g for 10 min. Supernatant (cytosolic fraction) was cleared by centrifugation at 10,000 g.

Nuclei and membranes were re-suspended in 1 ml of buffer B (buffer A supplemented with 0.5% NP-40 and 1 mM PMSF) and separated by passing the suspension again eight times through a 23-gauge needle followed by 20 rounds through a glass-glass homogenizer. The homogenate was centrifuged for 10 min at 900 g to pellet the nuclei. The supernatant (membrane fraction) was transferred to a fresh microfuge tube and again cleared by centrifugation at 10000 g for 15 min. The nuclear pellet was re-suspended in 1 ml of buffer C (buffer A containing 1 mM PMSF), and purity of nuclei was verified under a microscope. Resuspended nuclei were centrifuged at 1000 g for 10 min. The pellet was re-suspended in lysis buffer, sonicated briefly, and used for IP or prepared for Western blotting by addition of SDS-PAGE sample buffer. Alternatively, the nuclear enrichment kit from PIRCE was used to separate the nuclear fraction from membrane/cytosolic fraction according to manufacturer's protocol.

### Cellular growth assay and cell cycle analysis

For cellular growth assay, cells stably transduced (with retroviral constructs) or transiently transfected (with Lipofectamine 2000; Invitrogen) with constructs as indicated were plated in 96-well plates at 5×10^3^ cells/well. The cell number was determined 48 hr after plating based on the fluorescence intensity of SYBR green I nucleic acid staining (Molecular Probes, Eugene, OR) measured in a fluorescence microplate reader (Fluostar from BMG, Durham, NC). The data were expressed as mean ± SD from triplicate assays.

Cell cycle assay was performed using Guava cell cycle kit according to the manufacturer's protocol 48 hr after transient transfection or stable transduction of cell lines. Samples were analyzed in a Muse cell analyzer (EMD Millipore).

### Luciferase reporter assay

Cells were plated in 24-well plates and were transiently transfected (using Lipofectamine 2000) with 50 ng of pRL-Renilla, 0.5 µg of the Notch reporter pJH23A (4×wtCBF1Luc) luciferase construct (provided by D. Hayward, Johns Hopkins University), and 0.25 µg of various plasmid combinations as indicated in individual experiments. Assay analysis was carried out 24 h after transfection using the Dual-Glo Luciferase Reporter Assay (Promega) according to the manufacturer's protocol. Expression was normalized to pRL-Renilla Luciferase. The data were expressed as mean ± SD of triplicate assays.

### Bioinformatics analysis

Level 3 TCGA data on ovarian HGSC were retrieved from Broad Institute's Genome Data Analysis Center (http://gdac.broadinstitute.org/runs/stddata__2014_01_15/data/OV/20140115/). Data used include transcriptome on Affymetrix U133a platform (http://gdac.broadinstitute.org/runs/stddata__2014_01_15/data/OV/20140115/gdac.broadinstitute.org_OV.Merge_transcriptome__ht_hg_u133a__broad_mit_edu__Level_3__gene_rma__data.Level_3.2014011500.0.0.tar.gz) and somatic copy number alterations on Affymetrix SNP 6 platform (http://gdac.broadinstitute.org/runs/stddata__2014_01_15/data/OV/20140115/gdac.broadinstitute.org_OV.Merge_snp__genome_wide_snp_6__broad_mit_edu__Level_3__segmented_scna_minus_germline_cnv_hg19__seg.Level_3.2014011500.0.0.tar.gz).

#### Copy number analysis by GISTIC 2.0

GISTIC 2.0 (Genomic Identification of Significant Targets in Cancer) [15] was used to infer the significantly deleted/amplified genomic intervals across 569 primary ovarian HGSCs. The GISTIC algorithm calculated G-scores and q-values along the genomic positions, which reflected the significance of copy number alterations of given loci across all samples. The GISTIC output table (all_thresholded.by_genes.txt) was used to determine the putative copy-number status of WWP2 in each sample. The entries with value −2, −1, 0, 1, 2 were classified as homozygous deletion, hemizygous deletion, diploid, gain, and amplification, respectively.

#### Correlation between WWP2 mRNA expression and copy number alterations

To compare the distribution of mRNA expression among different copy-number status, we analyzed 554 ovarian HGSCs that had both putative copy-number status determined by GISTIC and transcriptome data on Affymetrix U133a platform. CNTools (v.1.18) were used to convert copy number segment data to gene-wise log2 copy number data. The transcriptome data were scaled as z-scores using the mean and standard deviation of those in samples with putative diploid status. The relationship between WWP2 DNA copy number and transcript expression was determined by Pearson correlation coefficient.

### Hoechst 33342 staining and detection of side population

Ovarian cancer cells were transfected with pLPC, pLPC-WWP2, SP-N3-NEXT or pLPC-WWP2+SP-N3-NEXT for 3 days, and cells were detached by trypsin, washed with PBS, then re-suspended in RPMI culture medium (supplemented with 2% FBS). The concentration of cells were adjusted to 1×10^6^ cells/ml and Hoechst 33342 (Sigma) was added at a final concentration of 5.0 µg/mL either alone or in the presence of 50 µg/mL verapamil. After incubation at 37°C for 90 min, cells were washed with ice cold PBS and then subjected to flow cytometry analysis. Hoechst 33342 was excited with UV laser at 350 nm and fluorescence emission was measured with 405/BP30 and 570/BP20 optical filters for Hoechst blue and Hoechst red emission wavelengths, respectively. A 550-nm long-pass dichroic mirror (Omega Optical Inc., Brattleboro, VT) was used to separate the emission wavelengths.

### Immunofluorescence staining

To visualize the subcellular localization of WWP2 and Notch3, MCF7 cells were seeded on gelatin-coated coverslips in a 6-well plate and treated with 25 mM NH_4_Cl for 2 hr and 4 hr or with 2.5 mM EDTA for 30 min. Cells were fixed with 3.7% paraformaldehyde/PBS for 10 min, permeabilized with 0.5% triton X-100/PBS for 5 min, and blocked with 1% normal goat serum for 30 min. Cells were incubated with an anti-Notch3 antibody (1∶100 dilution; Santa Cruz Biotechnology) followed by incubation with an anti-rabbit Cy3-conjugated antibody (1∶500 dilution; Sigma). After washing with PBS, cells were incubated with anti-WWP2 antibody (1∶100 dilution; Santa Cruz Biotechnology) and followed by anti-mouse Alex488-conjugated antibody (1∶500; Cell Signaling) for 1 hr. DNA was counterstained with DAPI (Invitrogen) for 1 min. Fluorescence images were acquired with a fluorescence microscope (TE200, Nikon).

### Gene knockdown and luciferase reporter assay

WWP2-specific small interfering RNAs (siRNAs) were purchased from Invitrogen. The target sequences of WWP2 siRNAs are: UAGACACGUCCGUUGGGCAGCUCUC and ACACGGGCUUCACCCUCCCUUUCUA. OSE4 and FT2821 cells were transfected with siRNAs at a final concentration of 100 nM using Lipofectamine RNAiMAX (Invitrogen) according to the manufacturer's protocol. Twenty-four hours after, cells were further transfected with 1 µg of Notch reporter plasmid, pJH23A (4×wtCBF1Luc), and 50 ng of pRL-Renilla for measuring the Notch signaling activity.

### Ovarian cancer xenograft model

To determine whether WWP2 may act as a tumor suppressor, tumorigenic carboplatin-resistant SKOV3 cells (SKOV3-CR) were transiently transfected with a plasmid encoding WWP2 cDNA or a control plasmid, pLPC. Six-week-old female athymic *nu/nu* mice were subcutaneously inoculated with 3×10^6^ tumor cells. Ten mice were included in each experimental group. Tumor size was measured every three days using a caliper and at the end of the study, tumors were carefully excised and weighted. Tumor volume based on caliper measurements were calculated by the modified ellipsoidal formula: Tumor volume = 1/2 (length×width×height).

## Supporting Information

Figure S1 Co-immunoprecipitation performed to detect ubiquitination of N3 fragments (A) and interaction between WWP2 and N3 fragments (B).
(PDF)

Figure S2 Localization of WWP2 and Notch3 in cancer cells treated with NH4Cl. MCF7 cells were treated with either NH4Cl (A) or EDTA (B) for the indicated time and cells were subjected to immunofluorescence staining. In NH4Cl treated cells, WWP2 and Notch3 protein fragments are colocalized. However, in EDTA treated cells, WWP2 and N3-ICD are located in different cellular compartments. As shown in Fig. 4, NH4Cl treatment resulted in release and cytoplasmic accumulation of N3-NEXT while EDTA treatment induces release and nuclear accumulation of N3-ICD.
(PDF)

Figure S3 GISTIC analysis of ovarian HGSC in TCGA dataset demonstrated significantly deleted sub-chromosomal regions. Vertical axis: GISTIC score (left) and q-value (right).
(PDF)

Figure S4 Relationship between the WWP2 gDNA copy number alteration and the transcript expression level using ovarian HGSC dataset in the TCGA.
(PDF)

Figure S5 Ectopic WWP2 expression reduces Notch signaling in primary cultures of ovarian cancer cells with high levels of Notch3 expression. (A) Primary cultures of ovarian cancer cells were transfected with a Notch luciferase reporter and were co-transfected with the WWP2 cDNA expression construct or control plasmid. The data are normalized to the same cell group transfected with a control plasmid. Reporter activities are reduced in the cell cultures with higher levels of Notch3 expression. The data obtained from OVCAR3 was included as a reference. (B) Correlations between Notch signaling activity upon WWP2 transfection and Notch protein expression in 17 primary cultures of ovarian cancer samples. Red dot represents data obtained from OVCAR3. R = −0.46, p<0.05 (one-tailed Pearson test).
(PDF)

Figure S6 Knockdown efficiency of WWP2 siRNA. OSE4 and FT2821 were transfected with two different WWP2 siRNAs (#1 and #2) or scrambled siRNA (siSCR). Forty-eight hours later, cells were harvested and subjected to Western blot analysis. GAPDH was included as a loading control.
(PDF)

Figure S7 WWP2 overexpression leads to cell cycle arrest in OVCAR3 and MCF7 cells. OVCAR3 and MCF7 cells were transfected with WWP2 expressing plasmid and control vector (pLPC) and cell cycle analysis was performed two days after transfection. Ectopic WWP2 expression leads to G2/M arrest in OVCAR3 (A) and G0/G1 arrest in MCF7 (B).
(PDF)

Figure S8 WWP2 counteracts Notch3-induced platinum resistance. (A) Western blot analysis shows expression of N3-NEXT (V5 tagged) and WWP2 (FLAG tagged) in OVCAR3 cells after transfection. (B) Notch3 overexpression leads to an increased cell viability in the presence of carboplatin, while WWP2 expression sensitized cells to carboplatin. When cells were co-transfected with N3-NEXT and WWP2, the carboplatin sensitivity restored to a level close to the control (pLPC) group.
(PDF)

Table S1 Notch3 protein interactome.
(PDF)

Table S2 Notch3 interactome networks.
(PDF)
