# Supplementary material for: Notch3 Interactome Analysis Identified WWP2 as a Negative Regulator of Notch3 Signaling in Ovarian Cancer
Source: PLoS Genet. 2014 Oct 30;10(10):e1004751. doi: 10.1371/journal.pgen.1004751 (PMC4214668; doi:10.1371/journal.pgen.1004751)
Supplement: Figure S1 — Co-immunoprecipitation performed to detect ubiquitination of N3 fragments (A) and interaction between WWP2 and N3 fragments (B). (PDF) [file pgen.1004751.s001.pdf]

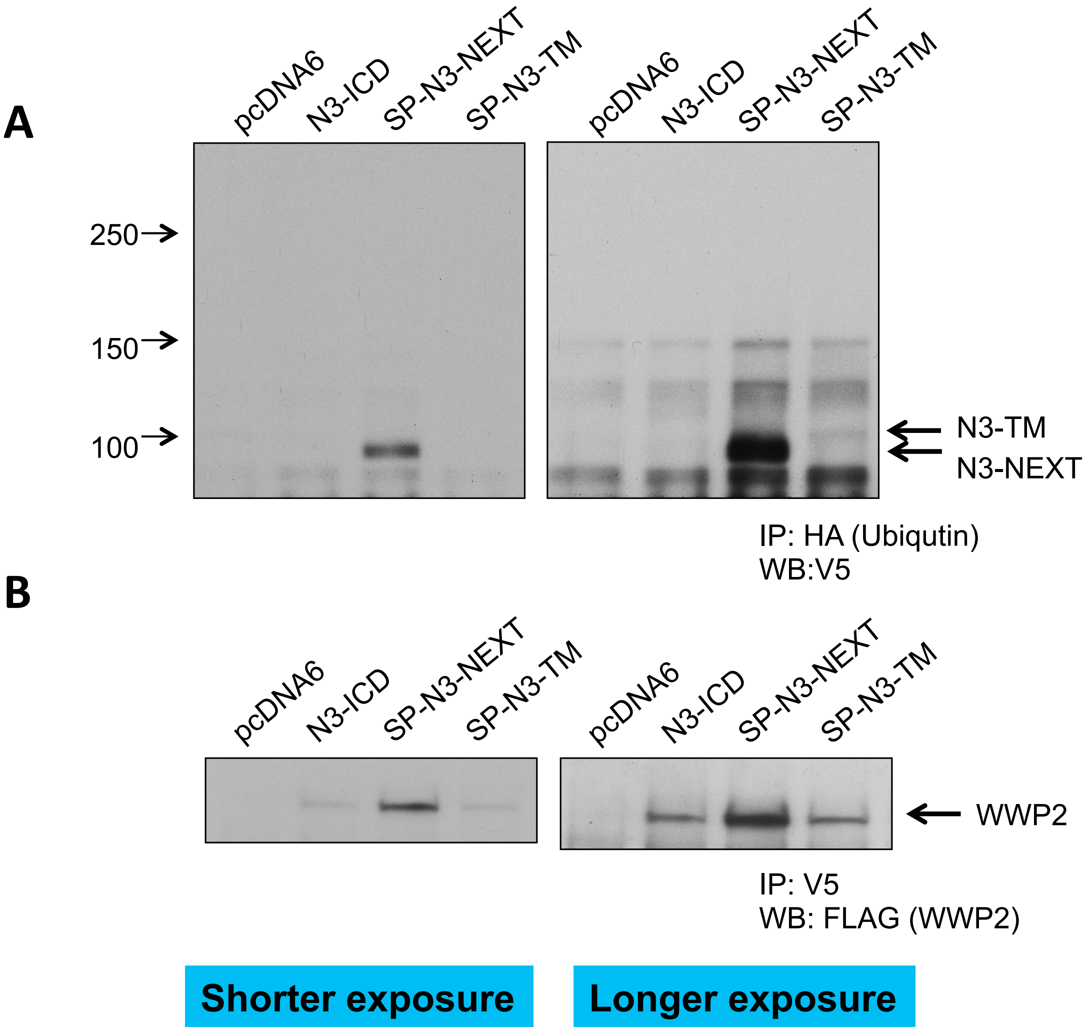

Fig. S1. Co-immunoprecipitation performed to detect ubiquitination of N3 fragments (A) and interaction between WWP2 and N3 fragments (B).
