# Supplementary material for: Notch3 Interactome Analysis Identified WWP2 as a Negative Regulator of Notch3 Signaling in Ovarian Cancer
Source: PLoS Genet. 2014 Oct 30;10(10):e1004751. doi: 10.1371/journal.pgen.1004751 (PMC4214668; doi:10.1371/journal.pgen.1004751)
Supplement: Figure S2 — Localization of WWP2 and Notch3 in cancer cells treated with NH4Cl. MCF7 cells were treated with either NH4Cl (A) or EDTA (B) for the indicated time and cells were subjected to immunofluorescence staining. In NH4Cl treated cells, WWP2 and Notch3 protein fragments are colocalized. However, in EDTA treated cells, WWP2 and N3-ICD are located in different cellular compartments. As shown in Fig. 4, NH4Cl treatment resulted in release and cytoplasmic accumulation of N3-NEXT while EDTA treatment induces release and nuclear accumulation of N3-ICD. (PDF) [file pgen.1004751.s002.pdf]

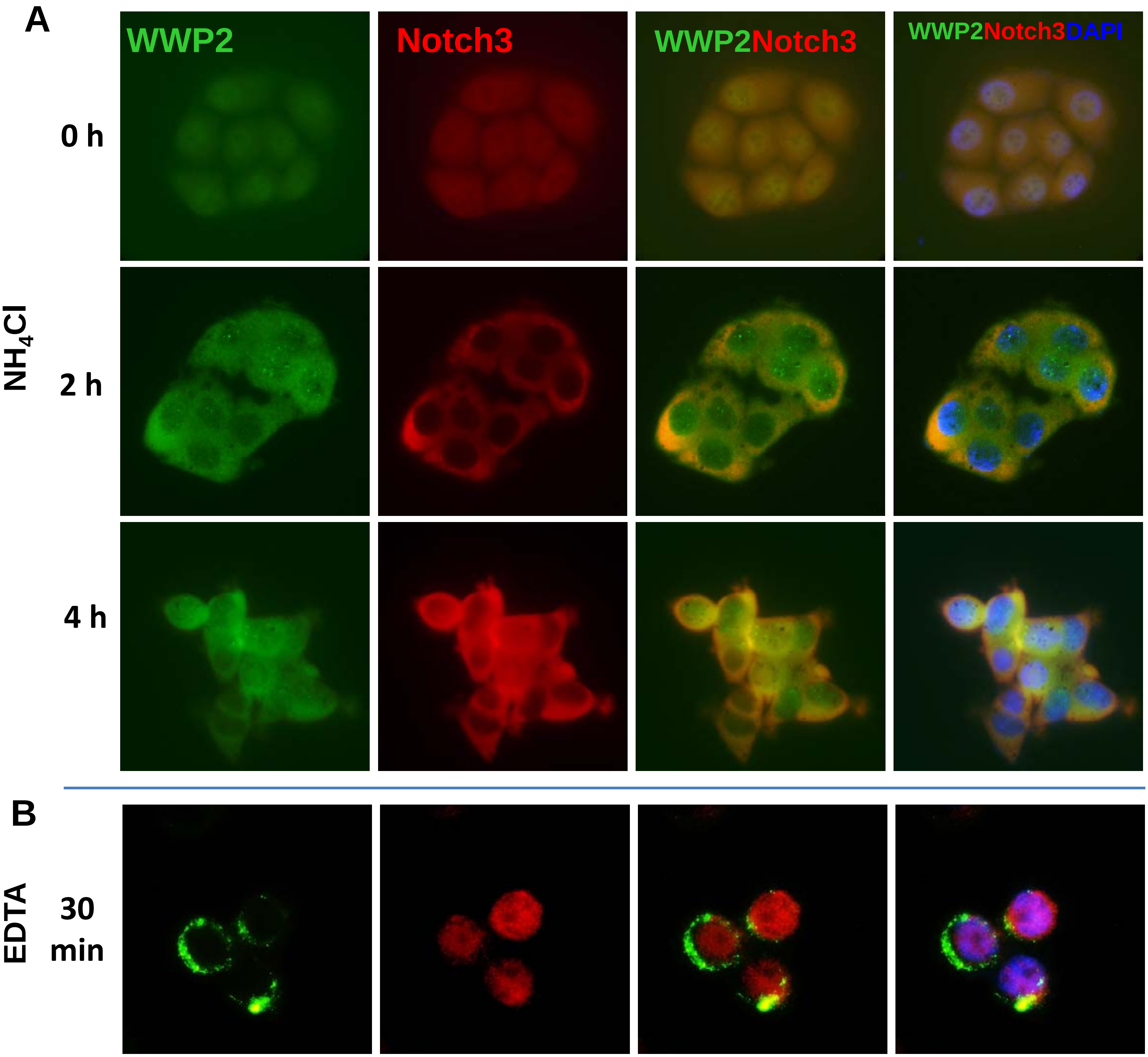

Fig. S2. Localization of WWP2 and Notch3 in cancer cells treated with NH_4Cl. MCF7 cells were treated with either NH_4Cl (A) or EDTA (B) for the indicated time and cells were subjected to immunofluorescence staining. In NH_4Cl treated cells, WWP2 and Notch3 protein fragments are co-localized. However, in EDTA treated cells, WWP2 and N3-ICD are located in different cellular compartments. As shown in Fig. 4, NH_4Cl treatment resulted in release and cytoplasmic accumulation of N3-NEXT while EDTA treatment induces release and nuclear accumulation of N3-ICD.
