# Supplementary material for: Notch3 Interactome Analysis Identified WWP2 as a Negative Regulator of Notch3 Signaling in Ovarian Cancer
Source: PLoS Genet. 2014 Oct 30;10(10):e1004751. doi: 10.1371/journal.pgen.1004751 (PMC4214668; doi:10.1371/journal.pgen.1004751)
Supplement: Figure S3 — GISTIC analysis of ovarian HGSC in TCGA dataset demonstrated significantly deleted sub-chromosomal regions. Vertical axis: GISTIC score (left) and q-value (right). (PDF) [file pgen.1004751.s003.pdf]

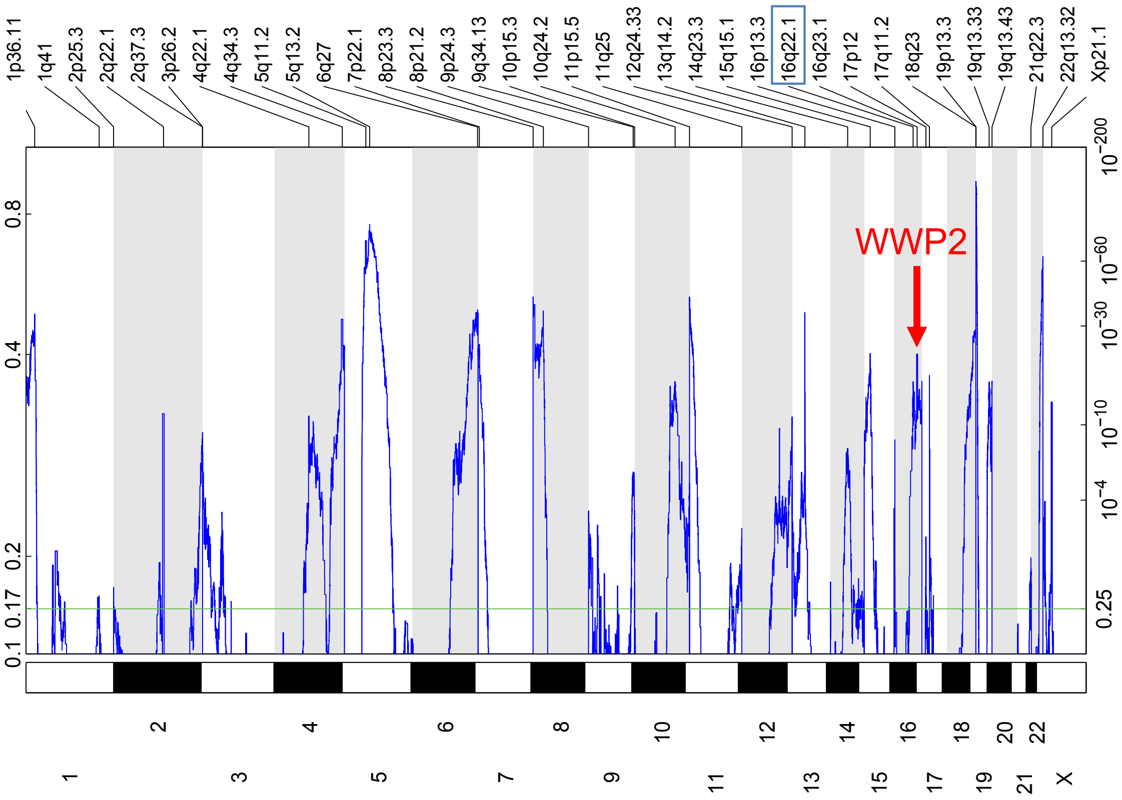

Fig. S3. GISTIC analysis of ovarian HGSC in TCGA dataset demonstrated significantly deleted sub-chromosomal regions. Vertical axis: GISTIC score (left) and q-value (right).
