# Supplementary material for: Notch3 Interactome Analysis Identified WWP2 as a Negative Regulator of Notch3 Signaling in Ovarian Cancer
Source: PLoS Genet. 2014 Oct 30;10(10):e1004751. doi: 10.1371/journal.pgen.1004751 (PMC4214668; doi:10.1371/journal.pgen.1004751)
Supplement: Figure S4 — Relationship between the WWP2 gDNA copy number alteration and the transcript expression level using ovarian HGSC dataset in the TCGA. (PDF) [file pgen.1004751.s004.pdf]

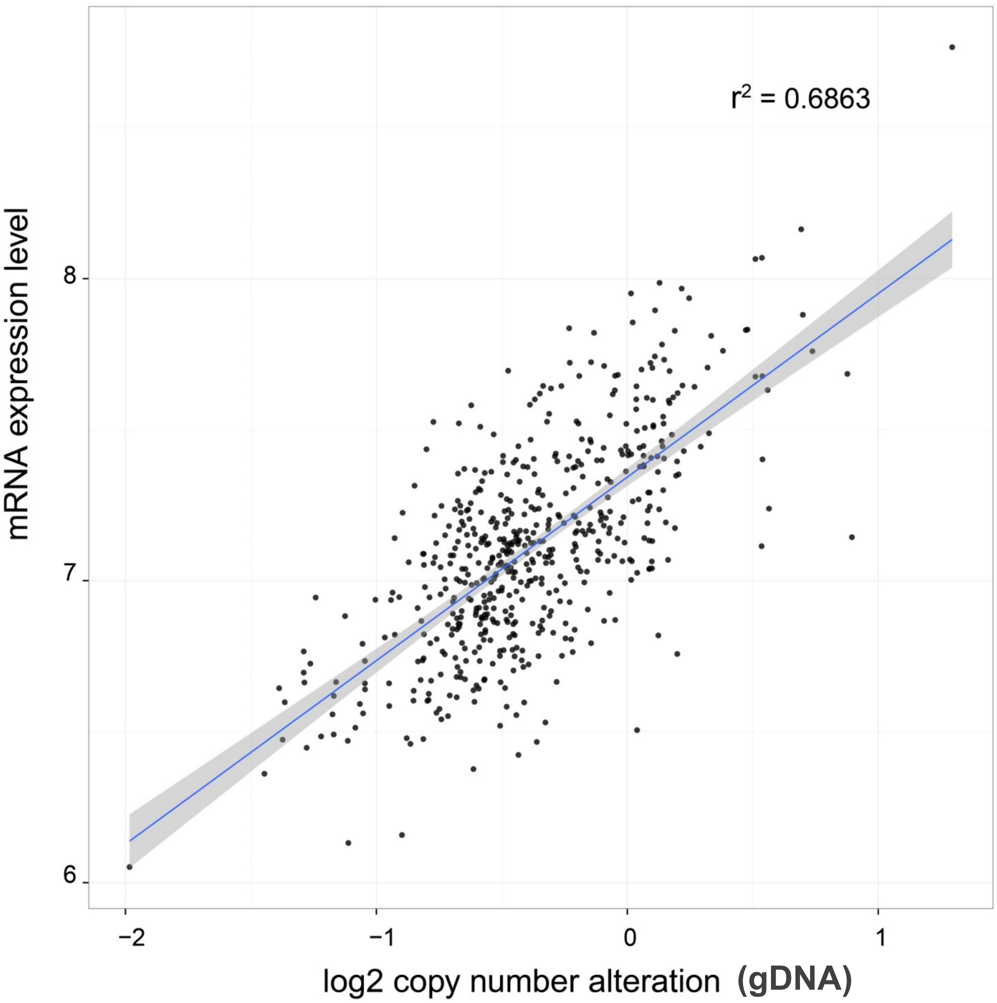

Fig. S4. Relationship between the WWP2 gDNA copy number alteration and the transcript expression level using ovarian HGSC dataset in the TCGA.
