# Supplementary material for: Notch3 Interactome Analysis Identified WWP2 as a Negative Regulator of Notch3 Signaling in Ovarian Cancer
Source: PLoS Genet. 2014 Oct 30;10(10):e1004751. doi: 10.1371/journal.pgen.1004751 (PMC4214668; doi:10.1371/journal.pgen.1004751)
Supplement: Figure S5 — Ectopic WWP2 expression reduces Notch signaling in primary cultures of ovarian cancer cells with high levels of Notch3 expression. (A) Primary cultures of ovarian cancer cells were transfected with a Notch luciferase reporter and were co-transfected with the WWP2 cDNA expression construct or control plasmid. The data are normalized to the same cell group transfected with a control plasmid. Reporter activities are reduced in the cell cultures with higher levels of Notch3 expression. The data obtained from OVCAR3 was included as a reference. (B) Correlations between Notch signaling activity upon WWP2 transfection and Notch protein expression in 17 primary cultures of ovarian cancer samples. Red dot represents data obtained from OVCAR3. R = −0.46, p<0.05 (one-tailed Pearson test). (PDF) [file pgen.1004751.s005.pdf]

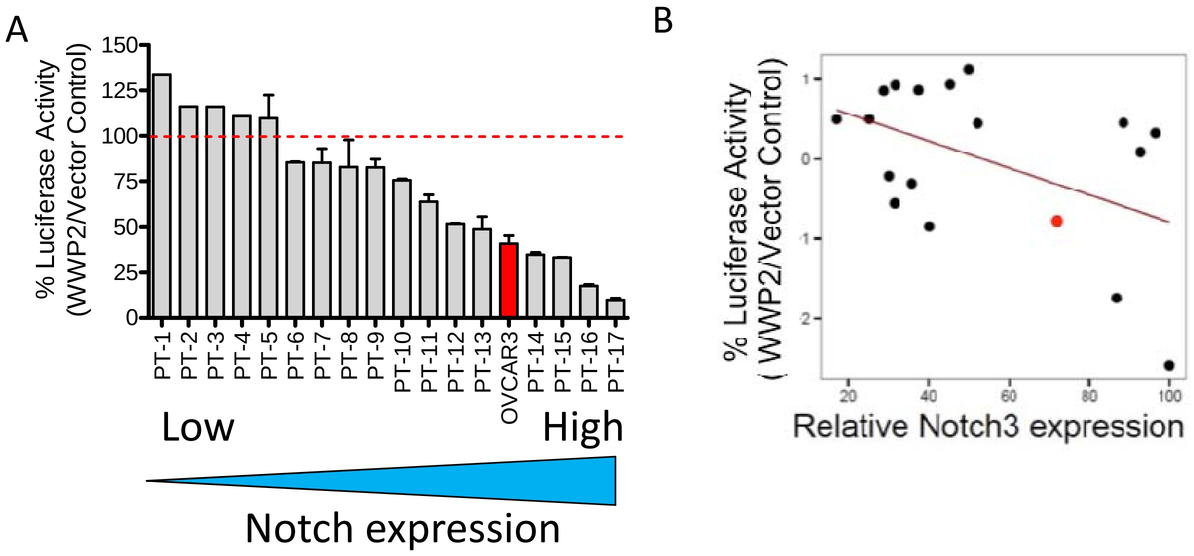

Fig. S5. Ectopic WWP2 expression reduces Notch signaling in primary cultures of ovarian cancer cells with high levels of Notch3 expression.

(A) Primary cultures of ovarian cancer cells were transfected with a Notch luciferase reporter and were co-transfected with the WWP2 cDNA expression construct or control plasmid. The data are normalized to the same cell group transfected with a control plasmid. Reporter activities are reduced in the cell cultures with higher levels of Notch3 expression. The data obtained from OVCAR3 was included as a reference. (B) Correlations between Notch signaling activity upon WWP2 transfection and Notch protein expression in 17 primary cultures of ovarian cancer samples. Red dot represents data obtained from OVCAR3. R= -0.46, $p < 0.05$ (one-tailed Pearson test).
