# Supplementary material for: Notch3 Interactome Analysis Identified WWP2 as a Negative Regulator of Notch3 Signaling in Ovarian Cancer
Source: PLoS Genet. 2014 Oct 30;10(10):e1004751. doi: 10.1371/journal.pgen.1004751 (PMC4214668; doi:10.1371/journal.pgen.1004751)
Supplement: Figure S6 — Knockdown efficiency of WWP2 siRNA. OSE4 and FT2821 were transfected with two different WWP2 siRNAs (#1 and #2) or scrambled siRNA (siSCR). Forty-eight hours later, cells were harvested and subjected to Western blot analysis. GAPDH was included as a loading control. (PDF) [file pgen.1004751.s006.pdf]

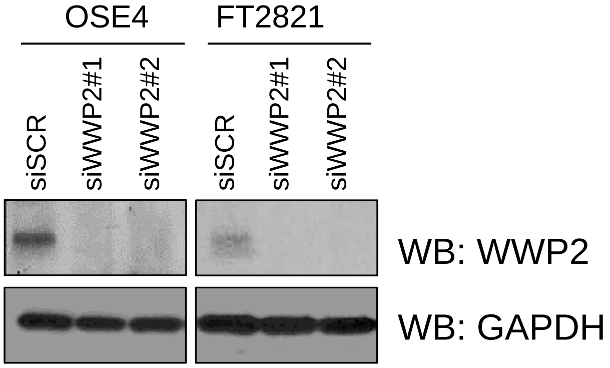

Fig. S6: Knockdown efficiency of WWP2 siRNA. OSE4 and FT2821 were transfected with two different WWP2 siRNAs (#1 and #2) or scrambled siRNA (siSCR). Forty-eight hours later, cells were harvested and subjected to Western blot analysis. GAPDH was included as a loading control.
