# Supplementary material for: Notch3 Interactome Analysis Identified WWP2 as a Negative Regulator of Notch3 Signaling in Ovarian Cancer
Source: PLoS Genet. 2014 Oct 30;10(10):e1004751. doi: 10.1371/journal.pgen.1004751 (PMC4214668; doi:10.1371/journal.pgen.1004751)
Supplement: Figure S7 — WWP2 overexpression leads to cell cycle arrest in OVCAR3 and MCF7 cells. OVCAR3 and MCF7 cells were transfected with WWP2 expressing plasmid and control vector (pLPC) and cell cycle analysis was performed two days after transfection. Ectopic WWP2 expression leads to G2/M arrest in OVCAR3 (A) and G0/G1 arrest in MCF7 (B). (PDF) [file pgen.1004751.s007.pdf]

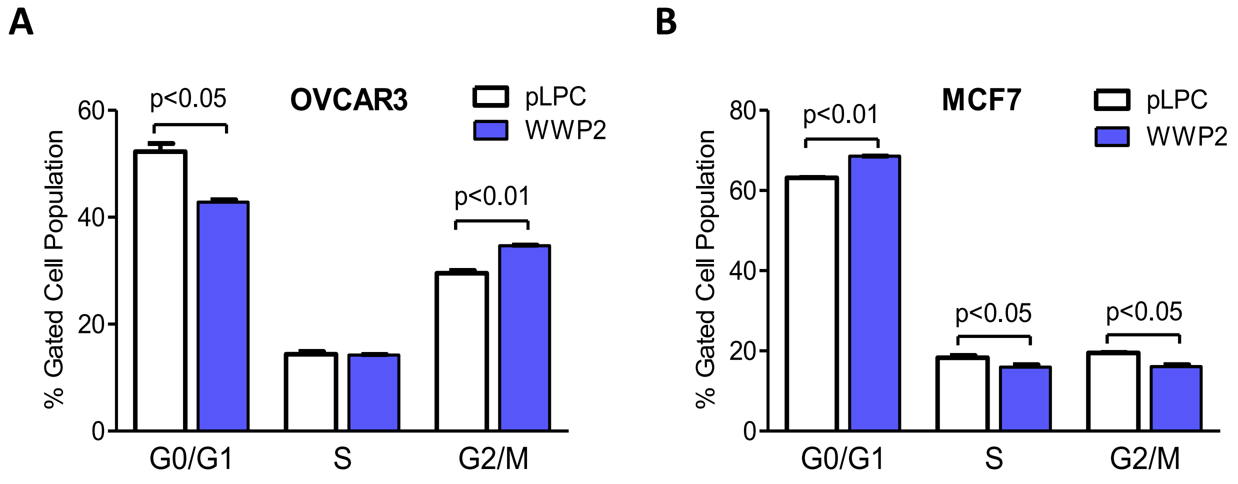

Fig. S7. WWP2 overexpression leads to cell cycle arrest in OVCAR3 and MCF7 cells. OVCAR3 and MCF7 cells were transfected with WWP2 expressing plasmid and control vector (pLPC) and cell cycle analysis was performed two days after transfection. Ectopic WWP2 expression leads to G2/M arrest in OVCAR3 (A) and G0/G1 arrest in MCF7 (B).
