# Supplementary material for: Notch3 Interactome Analysis Identified WWP2 as a Negative Regulator of Notch3 Signaling in Ovarian Cancer
Source: PLoS Genet. 2014 Oct 30;10(10):e1004751. doi: 10.1371/journal.pgen.1004751 (PMC4214668; doi:10.1371/journal.pgen.1004751)
Supplement: Figure S8 — WWP2 counteracts Notch3-induced platinum resistance. (A) Western blot analysis shows expression of N3-NEXT (V5 tagged) and WWP2 (FLAG tagged) in OVCAR3 cells after transfection. (B) Notch3 overexpression leads to an increased cell viability in the presence of carboplatin, while WWP2 expression sensitized cells to carboplatin. When cells were co-transfected with N3-NEXT and WWP2, the carboplatin sensitivity restored to a level close to the control (pLPC) group. (PDF) [file pgen.1004751.s008.pdf]

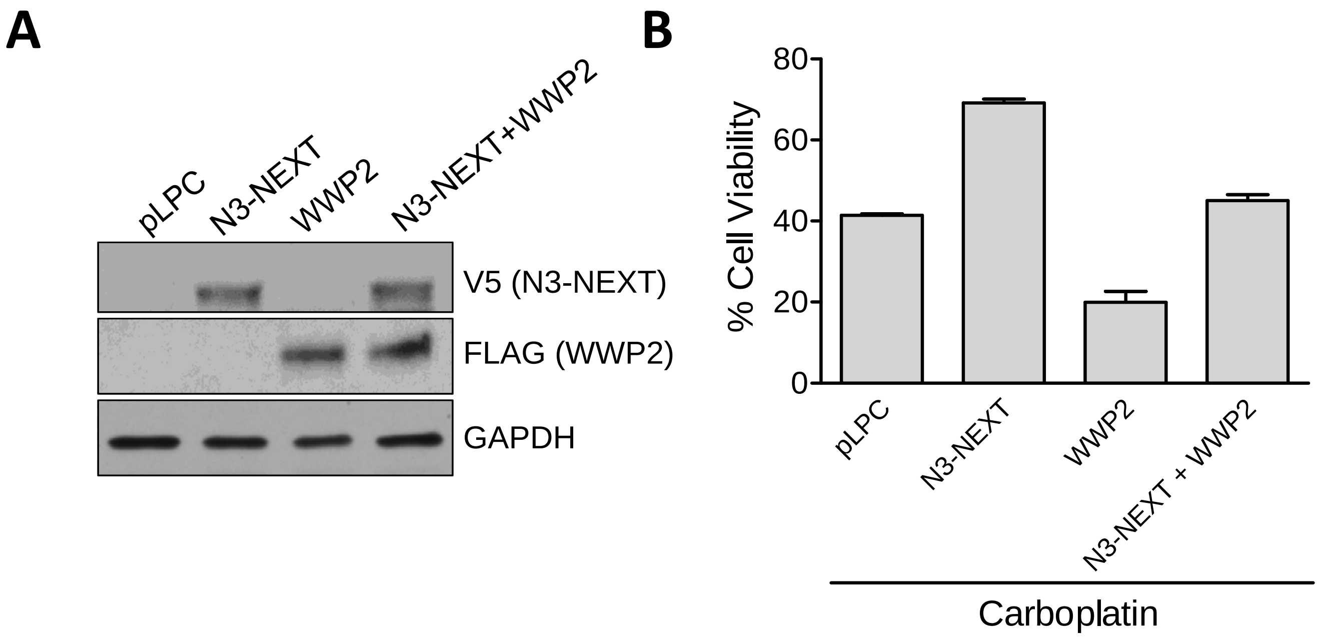

Fig. S8. WWP2 counteracts Notch3-induced platinum resistance. (A) Western blot analysis shows expression of N3-NEXT (V5 tagged) and WWP2 (FLAG tagged) in OVCAR3 cells after transfection. (B) Notch3 overexpression leads to an increased cell viability in the presence of carboplatin, while WWP2 expression sensitized cells to carboplatin. When cells were co-transfected with N3-NEXT and WWP2, the carboplatin sensitivity restored to a level close to the control (pLPC) group.
