# Supplementary material for: Notch3 Interactome Analysis Identified WWP2 as a Negative Regulator of Notch3 Signaling in Ovarian Cancer
Source: PLoS Genet. 2014 Oct 30;10(10):e1004751. doi: 10.1371/journal.pgen.1004751 (PMC4214668; doi:10.1371/journal.pgen.1004751)
Supplement: Table S1 — Notch3 protein interactome. (PDF) [file pgen.1004751.s009.pdf]

Table S1. Notch3 Protein Interactome

Name	ID	Intensity Score
SORBS3	IOH12305	61.6
WWP2	IOH4735	57.1
WWP2	IOH4735	47.6
SORBS3	IOH12305	46.4
RBPJ	IOH52060	35.1
RBPJ	IOH52060	32.2
TNNT2	IOH5297	24.6
TNNT2	IOH5297	24.4
XKR8	IOH4960	20.1
SNX19	IOH27386	19.6
CNTD1	IOH22486	19.4
XKR8	IOH4960	19.2
CNTD1	IOH22486	19.1
WDR25	IOH5172	18.6
SCGB3A1	IOH22643	18.4
CPNE6	IOH55460	17.6
CPNE6	IOH55460	16.3
WDR25	IOH5172	16.1
SLC39A1	IOH5139	15.8
BAG2	IOH40925	15.7
SCGB3A1	IOH22643	15.0
SLC39A1	IOH5139	14.9
PPIA	IOH59992	13.5
PPIA	IOH59992	13.5
GGT6	IOH40740	13.3
C8orf76	IOH40091	13.3
C1QL1	IOH6218	12.7
C1QL1	IOH6218	12.7
TMEM108	IOH4185	12.6
TMEM108	IOH4185	11.6
C16orf75	IOH23171	11.5
CRIP2	IOH3437	11.0
NEDD9	IOH27637	10.8
C16orf75	IOH23171	10.8
TPT1	IOH29340	10.5
CRIP2	IOH3437	10.2
SNX19	IOH27386	9.3
BTF3L4	IOH14732	9.0
RET	IOH5435	8.6

MPPED2	IOH22675	8.5
NEDD9	IOH27637	8.4
C8orf76	IOH40091	8.4
BAG2	IOH40925	8.4
CBFA2T2	IOH25743	8.3
BTF3L4	IOH14732	8.3
TPT1	IOH29340	8.3
MYC	IOH2954	8.2
MPPED2	IOH22675	8.2
GGT6	IOH40740	8.1
CBFA2T2	IOH25743	8.1
SPTLC2	IOH5616	7.5
RET	IOH5435	7.5
SPTLC2	IOH5616	7.4
MYC	IOH2954	7.0
