# Supplementary material for: Notch3 Interactome Analysis Identified WWP2 as a Negative Regulator of Notch3 Signaling in Ovarian Cancer
Source: PLoS Genet. 2014 Oct 30;10(10):e1004751. doi: 10.1371/journal.pgen.1004751 (PMC4214668; doi:10.1371/journal.pgen.1004751)
Supplement: Table S2 — Notch3 interactome networks. (PDF) [file pgen.1004751.s010.pdf]

Table S2. Notch3 Interactome Networks

Score	Focus Molecules	Top Functions	Molecules in the Network
38	15	Gene Expression, Cell Cycle, Hair and Skin Development and Function	ANXA7,betaestradiol,BTF3L4,C1QL1,C8orf76,CAPN15,CBFA2T2,CCND1,CNTD1,CPNE6,ERBB2,FOS,GGT6,GTF2H2D,KIAA0922,LOC391322,MPPED2,PRSS22,PSME3,RMI2,SLC13A3,SLC39A1,SNX19,SS18L2,TAPT1,TGFB1,TLDC1,TMEM108,TMEM150C,TMEM184B,TNNT2,UBC,WDR25,WWP2,ZNF75D
26	11	Cell Signaling, Cellular Development, Cellular Growth and Proliferation	Myc,CD3,CLEC4A,CRIP2,DOK5,ERK,ERK1/2,Fgf18,Immunoglobulin,LRRN1,Max-Myc,MYC,NEDD9,NFkB (complex),P38 MAPK,PPIA,RBPJ,RET,RFNG,SCGB3A1,Sh2b3,SORBS3,Sos,SPTLC2,STYX,TAS1R1,Tgf
